# Evolution and functional diversification of catalase genes in the green lineage

**DOI:** 10.1186/s12864-022-08621-6

**Published:** 2022-06-01

**Authors:** Luzhao Pan, Yin Luo, Jin Wang, Xiumin Li, Bingqian Tang, Huiping Yang, Xilin Hou, Feng Liu, Xuexiao Zou

**Affiliations:** 1 College of Horticulture, Nanjing Agricultural University, Nanjing, China; 2 College of Horticulture, Hunan Agricultural University, Changsha, China; 3 ERC for Germplasm Innovation and New Variety Breeding of Horticultural Crops, Changsha, China; 4 Key Laboratory for Vegetable Biology of Hunan Province, Changsha, China; 5 Longping Branch, Graduate School of Hunan University, Changsha, China

**Keywords:** Catalases, Green plants, Phylogeny, Functional diversification, Functionally conserved

## Abstract

**Background:**

Catalases (*CATs*) break down hydrogen peroxide into water and oxygen to prevent cellular oxidative damage, and play key roles in the development, biotic and abiotic stresses of plants. However, the evolutionary relationships of the plant CAT gene family have not been systematically reported.

**Results:**

Here, we conducted genome-wide comparative, phylogenetic, and structural analyses of *CAT* orthologs from 29 out of 31 representative green lineage species to characterize the evolution and functional diversity of CATs. We found that *CAT* genes in land plants were derived from core chlorophytes and detected a lineage-specific loss of *CAT* genes in Fabaceae, suggesting that the *CAT* genes in this group possess divergent functions. All *CAT* genes were split into three major groups (group α, β1, and β2) based on the phylogeny. *CAT* genes were transferred from bacteria to core chlorophytes and charophytes by lateral gene transfer, and this led to the independent evolution of two types of *CAT* genes: α and β types. Ten common motifs were detected in both α and β groups, and β *CAT* genes had five unique motifs, respectively. The findings of our study are inconsistent with two previous hypotheses proposing that (i) new *CAT* genes are acquired through intron loss and that (ii) the Cys-343 residue is highly conserved in plants. We found that new *CAT* genes in most higher plants were produced through intron acquisition and that the Cys-343 residue was only present in monocots, Brassicaceae and *Pp_CatX7* in *P. patens*, which indicates the functional specificity of the *CATs* in these three lineages. Finally, our finding that *CAT* genes show high overall sequence identity but that individual *CAT* genes showed developmental stage and organ-specific expression patterns suggests that *CAT* genes have functionally diverged independently.

**Conclusions:**

Overall, our analyses of the *CAT* gene family provide new insights into their evolution and functional diversification in green lineage species.

**Supplementary Information:**

The online version contains supplementary material available at 10.1186/s12864-022-08621-6.

## Background

The key components of the reactive oxygen species (ROS) gene network are thought to have originated as early as 4.1–3.5 billion years ago [1]. ROS-related proteins, such as catalases, are thought to have originated approximately 2.5 billion years ago, and the origin of these proteins was likely critically important to the survival of organisms during the Great Oxidation Event, which is thought to have occurred from 2.4 to 2.0 billion years ago [1]. After this planet-changing event, CATs evolved in aerobic organisms [2], which led to the appearance of three metalloenzyme families: (i) typical (monofunctional) heme CATs, (ii) (bifunctional) heme Catalase-Peroxidase, and (iii) (non-heme) manganese CATs [3–5]. The typical (monofunctional) heme CATs are most widespread in living organisms [6], they are the most extensively studied. Below, we summarize current research on typical CATs. Non-heme manganese CATs are widely distributed in microbial life and play an important role in antioxidant defense [7]. The typical heme CATs (hydrogen peroxide oxidoreductase E.C. 1.11.1.6) are critically important antioxidant enzymes that catalyze the degradation of hydrogen peroxide to produce water and oxygen [8]. CATs are mainly present in peroxisomes, but they have also been detected in the cytoplasm, mitochondria, and chloroplasts [9]. Typical heme catalases are the only CATs present in plants [2, 3, 8]. But in some plants (such as *Adiantum capillus-veneris* and *Marchantia polymorpha*), those CATs were generally classified into a distinct phylogenetic clade [8]. Unlike animals which only possess a single CAT, plants generally have multiple CAT isozymes [10], which suggests that the diversity in the structure and function of CATs among plants might be particularly rich [6, 10–13].

CATs play a key role in the development, defense, and senescence of plants, and several factors, such as hydrogen peroxide, temperature, plant hormones, wounding, and circadian rhythm, affect the expression of *CAT* genes [10, 13]. Plant *CATs* have been classified into three classes according to their expression properties, class I, class II, and class III, which are expressed in photosynthetic, vascular, and reproductive tissues, respectively [6]. In maize, *CAT-I* is transcribed and translated following tetrad formation and is expressed in mature pollen [14]; the expression of *Cat3* is regulated by circadian rhythm [15]. Recent studies have shown that maize *CATs* can facilitate the replication of maize chlorotic mottle virus [16]; catalase 1 in particular can promote viral multiplication and infection [17]. In *Arabidopsis*, *CATs* play key roles in the responses to irradiance and pathogens [18], signal transduction [19], plant hormones, plant senescence, and reproduction [20, 21]. The *Arabidopsis* CPK8 can phosphorylate the Ser-261 residue of CAT3 to regulate ABA-mediated stomatal regulation in response to drought stress [19]. The *cat1/2/3* triple mutant generated using CRISPR/Cas9 technology displays severe redox perturbation and growth defects [21]. In pepper, the expression of *CaCat1* and *CaCat2* is differentially regulated by circadian rhythm, and the expression of *CaCat1* responds to wounding and paraquat treatment [12].

A phylogenetic analysis of prokaryotes and eukaryotes revealed that *CATs* comprise three main clades: clade 1, clade 2, and clade 3 [2, 3, 8]. Clade 1-type *CATs* occur in eubacteria, algae, and plants; clade 2-type *CATs* occur in eubacteria and fungi; and clade 3-type *CATs* are the most abundant and have been detected in archaebacteria, fungi, protists, plants, and animals [2, 3, 8]. Clade 3-type *CATs* have been extensively studied in humans and other animals for their scientific and medical importance [2]. Clade 1-type *CATs* and clade 3-type *CATs* evolved from the older clade 2-type *CATs* [3, 8]. Plants generally contain both clade 1 and clade 3-type *CATs* [8]. Clade 1-type *CATs* are abundantly distributed in plants, ranging from unicellular green alga (*C. reinhardtii*) [22] to various land plants [11–13, 19]. A phylogenetic tree of 200 typical catalases were classified into three main evolutionary clades, and clade1 contained plant catalases major group, Firmicutes group A and Proteobacterial minor group [8]. Trees of 70 typical catalases from all main living kingdoms classify bacterial *CAT*s and major plant *CAT* members as clade 1 [2]. By contrast, clade 3-type *CATs* have only been detected in a few plant species to date [8]. Whether clade 3-type *CATs* are more widespread in plants and how these *CATs* evolved require further investigation. Plants also often possess multiple copies of *CAT* genes, but the evolutionary relationships among these genes have not yet been fully clarified.

Here, we conducted genome-wide comparative, phylogenetic, and structural analyses of 82 *CAT* sequences from major green lineage (green algae and land plants) taxa to evaluate the origin, distribution and duplication patterns, and functional features of *CAT* genes in plants. The results of our analyses revealed gene number variation in *CAT* genes among species and the lineage-specific loss of *CAT* genes in Fabaceae. *CAT* genes were divided into three subfamilies, and lateral gene transfer (LGT) led to the evolution of two clades of *CAT* genes. Paralogs were conserved among species, and changes in key amino acid residues might have led to the acquisition of new functions. The results of our study provide new insights into the evolution and functional diversification of *CATs* in plants.

## Results

### Homolog searches and verification of *CAT* genes

A BLASTP search was performed using the HMMER model to identify *CAT* genes among species with 31 fully sequenced genomes (Table 1). No *CAT* sequences were searched in *M. pusilla* and *O. lucimarinus,* so these two species were excluded. A total, 83 homolog sequences were identified from four algae (including three core chlorophytes and three charophytes), three bryophytes (liverworts, mosses and hornworts), one lycophyte, one gymnosperm, two ferns and 16 angiosperms (Table 1, Table S1). The Pfam database was then used to verify the presence of two conserved CAT domains in the putative CAT candidates: Catalase (PF00199.19) and Catalase-related immune-responsive (PF06628.12). These two domains were present in all 83 sequences and were thus used in subsequent analyses.

**Table 1:** The detail information of plant genome

Species name	Genome version	Gene number	Genome size	Gene number vs. genome size
*Micromonas pusilla*	CCMP1545 v3.0	0	22 Mbp	0
*Ostreococcus lucimarinus*	v2.0	0	13.2 Mbp	0
*Dunaliella salina*	v1.0	1	343.7 Mbp	0.29%
*Chlamydomonas reinhardtii*	v5.6	1	121 Mbp	0.83%
*Volvox carteri*	v2.1	1	131.2 Mbp	0.76%
*Mesostigma viride*	CCAC 1140	1	329 Mbp	0.30%
*Klebsormidium flaccidum*	v1.1	5	117.1 ± 21.8 Mbp	4.27%
*Chara braunii*	Cbr_1.0	1	1430 Mbp	0.069%
*Physcomitrella patens*	v3.3	8	473 Mbp	1.69%
*Marchantia polymorpha*	v3.1	4	225.8 Mbp	1.77%
*Anthoceros punctatus*		8	132.8 Mbp	6.02%
*Selaginella moellendorffii*	v1.0	3	212.5 Mbp	1.41%
*Azolla_filiculoides*	v1.2	2	759 Mbp	0.27%
*Salvinia cucullata*	v1.2	2	250 Mbp	0.80%
*Gnetum montanum*	v1.1	2	4200Mbp	0.05%
*Ananas comosus*	v3	3	526 Mbp	0.57%
*Brachypodium distachyon*	v3.1	3	272 Mbp	1.10%
*Oryza sativa*	v7.0	3	430 Mbp	0.70%
*Zea mays*	RefGen_V4	3	2300 Mbp	0.13%
*Sorghum bicolor*	v3.1.1	3	818 Mbp	0.37%
*Nicotiana plumbaginifolia*	NT	3	2000Mbp	0.15%
*Capsicum annuum*	cv CM334 _1.55	3	3070 ~ 3480Mbp	0.10%
*Solanum lycopersicum*	ITAG4.0	3	900 Mbp	0.33%
*Solanum tuberosum*	v4.03	3	850 Mbp	0.35%
*Capsella rubella*	v1.1	3	219 Mbp	1.37%
*Arabidopsis lyrata*	v2.1	3	207 Mbp	1.45%
*Arabidopsis thaliana*	TAIR10	3	125 Mbp	2.40%
*Glycine max*	Wm82.a2. v1	4	1115 Mbp	0.36%
*Cicer arietinum*	v1.0	1	738 Mbp	0.14%
*Trifolium pratense*	v2	1	420 Mbp	0.24%
*Medicago truncatula*	Mt4.0v1	1	454 Mbp	0.22%

*NT *Unknown

**Fig. 1:**
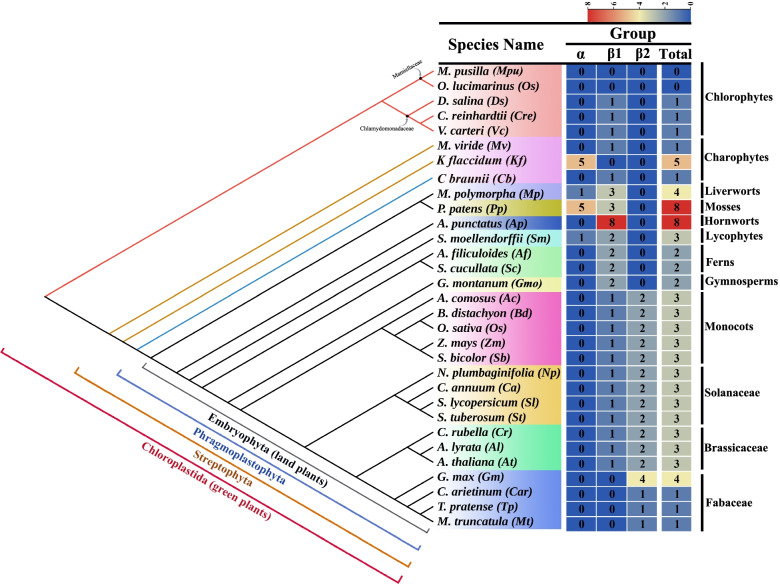
Number of CAT paralogs in each species and their distributions. The species tree is manually corrected with information on the TIMETREE website (http://timetree.org/)

One gene from *M. truncatula* (*Medtr1386s0010*) showed low sequence identity, and its position in the phylogeny, its intron phase, and motif elements were inconsistent with the general evolutionary patterns revealed by phylogenetic and structural analyses. Moreover, *C. arietinum* and *T. pratense*, which are from the same family as *M. truncatula*, only possessed a single CAT member (Fig. 1, Table S1). We speculate that *Medtr1386s0010*, which is categorized as encoding a CAT heme-binding enzyme in the NCBI database, was incorrectly annotated; this gene was thus excluded from subsequent analyses. The remaining 82 genes were renamed using the same nomenclature that has been used for *CAT* genes in previous studies of various taxa, such as *Arabidopsis*, rice, and maize. Published gene names were used for *CAT* genes that have been previously characterized and the remaining genes were sequentially arranged based on the abbreviation of the species name.

Information on the renamed *CATs* is shown in Table S1. The protein length of *CATs* varied substantially among core chlorophytes, charophytes, and lower land plants. Core chlorophytes *CAT* proteins all possessed 493 amino acid residues. *M. viride* and *C. braunii* all possessed 492 amino acids, whereas the length of *K. flaccidum* varied from 503 to 651 amino acids. The length of *CAT* proteins of some land plants (including bryophytes, lycophytes and ferns) was very unstable, ranging from 329 to 601 amino acid residues. On the contrary, the length of the *CATs* in the remaining plants was generally 492 amino acid residues; some minor sequences deviating from this pattern were likely caused by genome annotation errors. Some protein sequences were successfully annotated using online tools (Table S2).

### Gene number variation of *CAT* genes among various species

In total, orthologous *CAT* genes were identified from 29 out of 31 organisms, including six algas and 23 land plants (Fig. 1). Gene number variation (GNV) of *CAT* genes varied substantially among all organisms sampled, including core chlorophytes (one gene), *M. viride* and *C. braunii* (one), *K. flaccidum* (five), monocots (three), dicots (one to four), *A. punctatus* and *P. patens* (eight) (Table 1 and Fig. 1). These results suggest that there was no correlation between CAT gene number and genome size (Table 1).

GNV among species was mainly driven by differences among groups. Multiple *CAT* members from *K. flaccidum* and *P. patens* were observed in group α, and several *CAT* from bryophytes, lycophytes, ferns and gymnosperms were detected in group β1. Only *CAT* genes from angiosperms were included in group β2. Three *CAT* genes were present in all angiosperms, with the exception of members of Fabaceae, which only possessed a single *CAT* member; however, four *CATs* were observed in soybean.

No *CAT* genes were detected in Mamiellaceae (*M. pusilla* and *O. lucimarinus*), and only one *CAT* gene was detected in each member of the Chlamydomonadaceae (core chlorophytes) (Fig. 1), suggesting that *CAT* genes in plants might be derived from ancestral core chlorophyte genes. Multiple *CAT*s were detected in *K. flaccidum*, suggesting that the first large-scale expansion of *CAT* genes occurred in Charophyte.

### Classification of the *CAT* gene family in plants

**Fig. 2:**
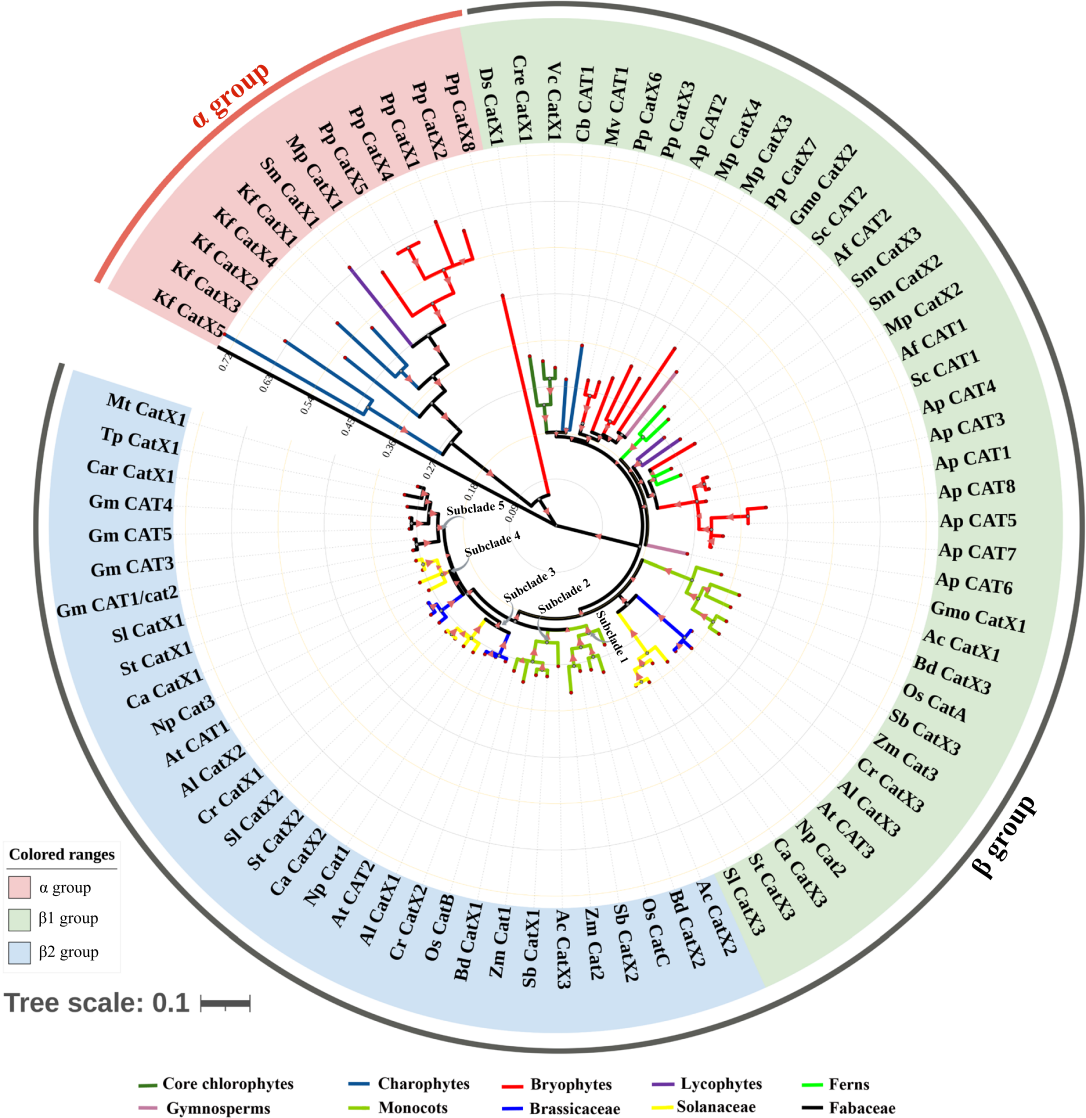
Phylogenetic relationship and classification of CAT genes from green algae to higher plants. The bar scale shows denote the number of amino acid replacements per site. Group α, β1 and β2 are represented by red, green and blue lines, respectively

A phylogenetic analysis was conducted using the full-length protein sequences from all organisms sampled with the ML and NJ methods to clarify the evolutionary relationships among *CAT* genes. The overall topologies of the ML and NJ trees were similar. Thus, only the ML tree was shown. All *CAT* genes could be divided into three major phylogenetic lineages, which were referred to as group α, β1 and β2 based on phylogeny (Fig. 2, Fig. S1). Meanwhile, this phylogenetic separation was supported by their different exon–intron structure (Fig. S2). Group α only included *CAT* genes from Charophyte, Bryophyte, and Lycophyte; Group β1 contained *CAT* genes from all green plants except for *K. flaccidum* and Fabaceae; and group β2 only has *CAT* genes from monocots and dicots.

All *CAT* genes from *K. flaccidum* were included in group α and have undergone at least two rounds of duplication (Fig. 2). A part of *CAT*s from bryophytes and lycophytes were also included in group α, but their evolutionary history was difficult to infer because of their high levels of sequence divergence. No *CAT*s from flowering plants were present in this group.

The core chlorophytes *CAT* genes were the earliest diverging lineage in group β1 and formed a small monophyletic cluster (Fig. 2). Multiple *CAT* genes from bryophytes and a lycophyte successively diverged, and at least one duplication event has occurred in each species. A single gymnosperm *CAT* gene and multiple *CAT* orthologs from angiosperms formed three distinct clades (Fig. 2). The phylogeny, coupled with gene structure analysis (Fig. S2), revealed substantial differences in the structure of *CAT* genes within group β1. By contrast, *CAT* orthologs within the same lineage, such as *CAT* orthologs within Poaceae, Brassicaceae, and Solanaceae, were relatively conserved in their intron phases, exon length, and number of exons (Fig. S2).

Group β2 was an angiosperm-specific clade and consists of five subclades. *CAT* genes underwent several duplication events prior to the divergence of monocots and dicots, indicating that monocots and dicots possess the ancestral genes in this clade. Within monocots, all *CAT* orthologs underwent a round of duplication, which resulted in the formation of subclades 1 and 2. Within dicots, *CAT* orthologs from rosids and asterids underwent a round of duplication, which generated subclades 3 and 4. *CAT* genes from the Fabaceae family appeared to be differentiated from those of other dicots, which resulted in the formation of the Fabaceae-specific subclade 5 with 86% bootstrap support.

### The evolutionary relationships of plant *CAT* gene family among the major lineages of Life

**Fig. 3:**
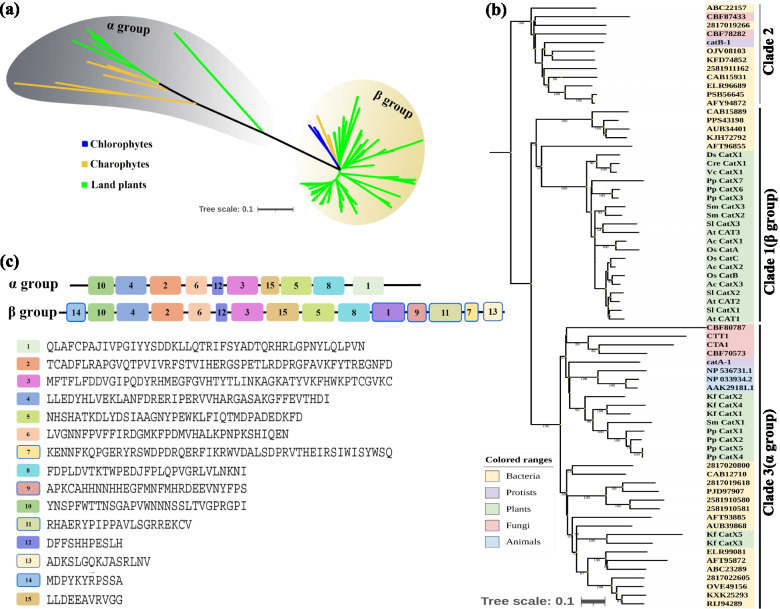
Origin and evolutionary trajectories of the CAT gene family in different organisms. (**a**) Cladogram of CATs from 29 green plants. The bar scale shows denote the number of amino acid replacements per site. (**b**) Phylogeny of CAT genes from representative bacteria, protists, fungi, plants, and animals. The bar scale shows denote the number of amino acid replacements per site. (**c**) Distribution of motifs 1 to 15 in the CAT gene family

Phylogenetic and structural analysis provided many insights into the origin and evolution of *CAT* genes. In Fig. 3a, group α and β can be treated as two distinct evolutionary types of CAT genes, respectively. The α group *CAT* genes evolved more rapidly than β *CAT*s according to the positions of their branch nodes and branch lengths. Furthermore, the α group was derived from *K. flaccidum* (charophyte), whereas the β group, which included all *CAT* genes from subfamilies β1 and β2, was derived from chlorophyte. In order to explore the evolutionary relationships of plant *CAT* genes among the major lineages of Life, we then constructed another phylogenetic tree using *CAT* sequences from representative bacteria, protists, fungi, plants, and animals to further trace the origin and evolution of *CAT* genes (Fig. 3b, Table S3). The phylogen revealed that the α and β groups of the CAT gene family corresponded exactly to clade 3 and clade 1 proposed by Zámocky [6], whereas clade 2 was a missing clade in the green lineage. Further analysis revealed that α and β *CAT* genes, both of which included *CAT* genes from bacteria with strong support, shared a common ancestral gene (Fig. 3b). The clade2 catalases were detected only in bacteria, protists and fungi. Clade 3 (α group) was widely distributed in bacteria, protists, fungi, animals and partial green plants, and clade1 (group β) was present in almost all analyzed plants (Fig. 3b). Group α and β shared 10 conserved motifs, but showed highly divergence at C-terminus and N-terminus (Fig. 3c).

### Key amino acid residues in Brassicaceae and monocots

**Fig. 4:**
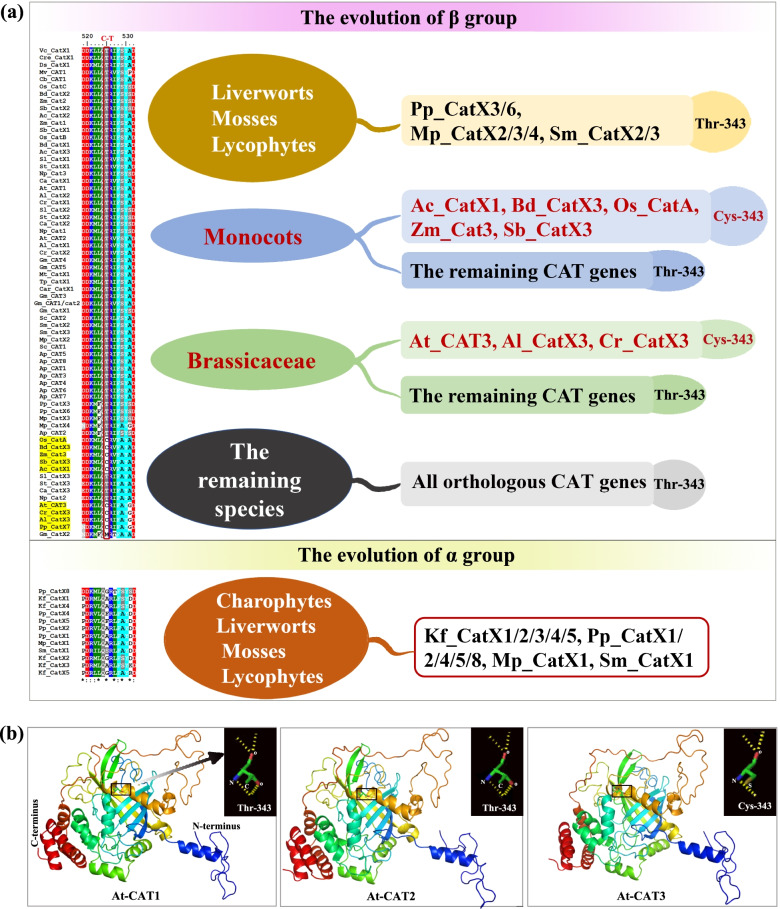
(**a**) Alignment and distribution of key amino acids residues for CAT proteins. The transition between residues C and T is marked above the sequence alignment. The distribution of T/C residues from distinct species is displayed in the right side. (**b**) Homolog models of At_CAT1, At_CAT2 and At_CAT3. Dashed yellow lines represent hydrogen bonds

A recent study has shown that CAT3 in *Arabidopsis* (i.e., ROG1) possesses a conserved Cys-343 residue that can decrease its catalase activity but increase its transnitrosylase activity [23]. Conversely, CAT2 (*At_CAT2*), which possesses a Thr-343 residue, shows reduced CAT activity but increased transnitrosylase activity [23]. Multiple sequence alignment of *CAT* sequences from β group (Fig. 4a, Fig. S4) revealed that the Cys-343 residue was only present in β1 group *CATs* from monocots and Brassicaceae. With the exception of *Gm_CatX2*, the remaining *CATs* were consistent with *At_CAT2* in *Arabidopsis* in possessing the highly conserved Thr-343 residue. It's worth noting that *Pp_CatX7* also contained a Cys-343 residue, but two *Mp_CatX3/4* orthologs, which were closely related to *Pp_CatX7* in the phylogenetic tree, had a Thr-343 residue instead of a Cys-343 residue. In addition, α *CATs* possessed neither a Cys-343 residue nor Thr343 residue (Fig. 4a, Fig. S4).

The three-dimensional structures of three *CATs* in *Arabidopsis* were predicted using Phyre2 Server to explore the structural characteristics of *CATs*. The high coverage and 100% confidence indicated that the modeling results were robust (Table S4). The prediction results indicated that these three genes have highly similar α-helices and β-strands (Fig. 4b). The Thr-343 residue of At-CAT1/2 possesses more atoms capable of forming hydrogen bonds than the Cys-343 residue of At-CAT3. The Cys-343 residue lacks the oxygen atoms needed to form hydrogen bonds; it also has a sulfur atom in the place of the carbon atom on the Thr-343 residue (Fig. 4b).

### High sequence identity among angiosperm *CAT* genes

**Fig. 5:**
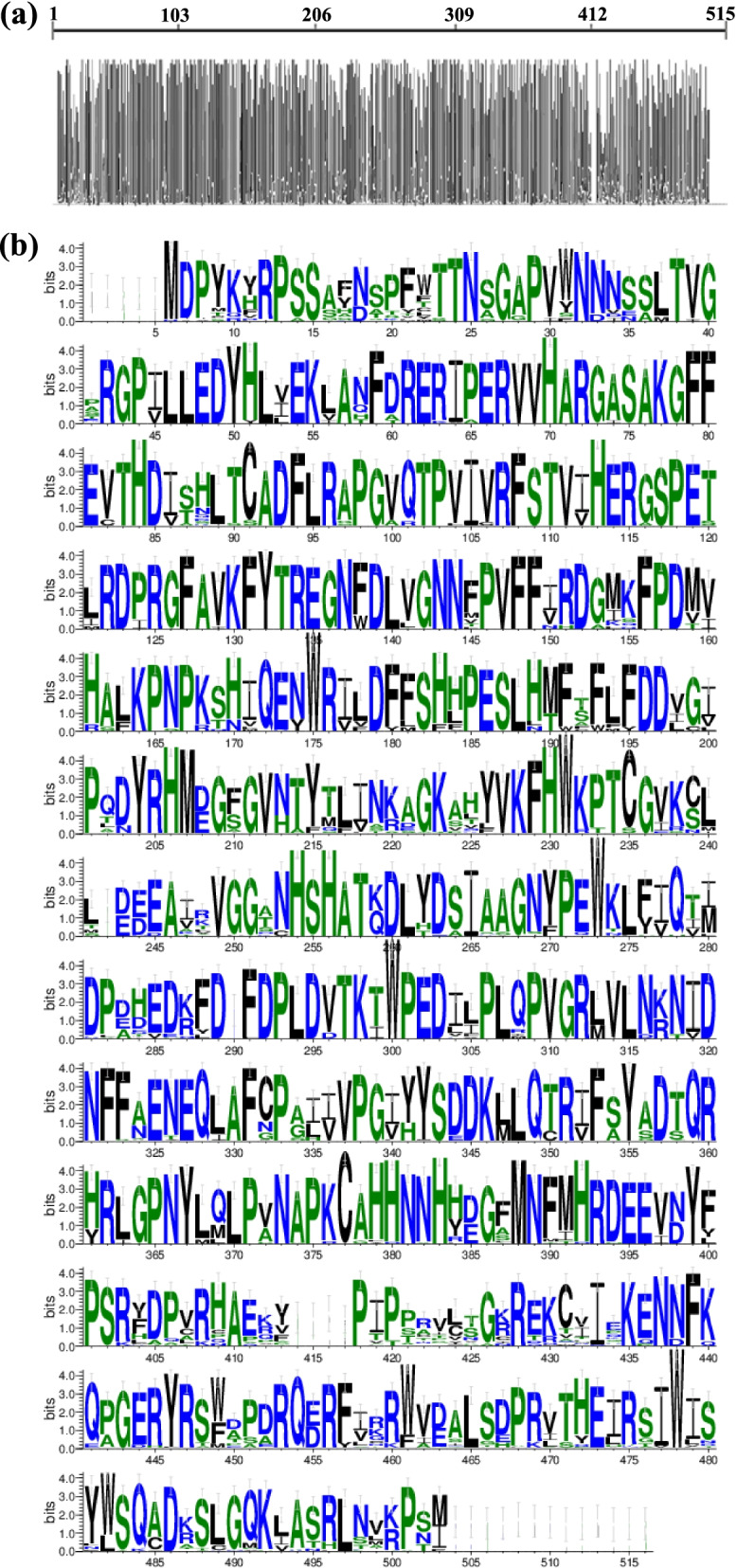
Multiple sequence alignments for CATs in angiosperms. (**a**) Amino acid conservationpatterns of CATs. (**b**) Sequence features of web logo in the CAT gene family

Multiple sequence alignments of *CAT* genes were performed to determine the degree of conservation and divergence in *CAT* genes among angiosperms. There was a high average pairwise identity (83.01%) ranging from 66.50% to 99.40% among angiosperm *CAT* genes (Fig. 5, Table S5). Extremely high sequence identity was observed among paralogs in individual species. For example, the paralogs of two monocots, *A. comosus* and *B. distachyon*, showed 90.04% and 86.04% sequence identity, respectively, and the paralogs of the dicots tobacco and *Arabidopsis* showed 88.28% and 89.02% sequence identity, respectively (Table S6). The highest sequence identity among paralogs was observed for soybean (94.82%) (Table S7).

### Gene duplication promoted increased expression and functional divergence in dicots

**Fig. 6:**
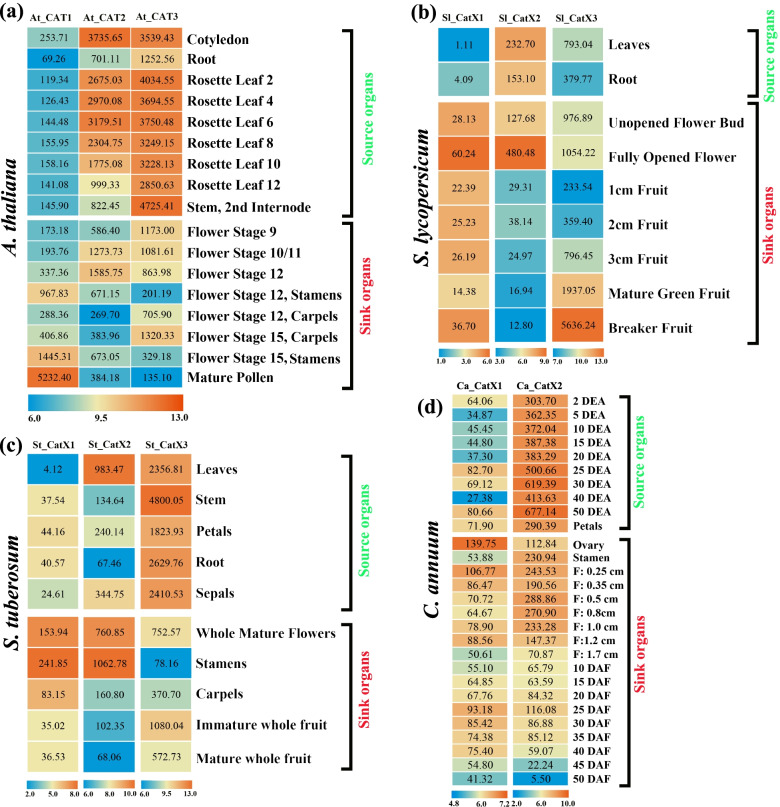
Expression patterns of CAT genes in four dicots. (**a**) A. thaliana, (**b**) S. lycopersicum (**c**) S. tuberosum and (**d**) C. annuum. The expression pattern of pepper Ca_CatX3 not included due to the lack of transcriptome data. The different tissues used for analysis were displayed on the right side of each corresponding heatmap, and gene name were shown above each column. All values were visualized as colored boxes, with blue, yellow and red indicating low, medium and high expression, respectively

Changes in the expression of genes can lead to changes in gene function, and these changes might in turn affect the growth and development of plants [24]. RNA-seq datasets from *Arabidopsis*, tomato, potato, and pepper were downloaded and analyzed to determine the possible functions of *CAT* genes in plants. Nearly all genes were expressed in all the tissues examined (Fig. 6). *CAT* genes exhibited developmental stage and organ-specific expression patterns in each species. Clade II-type *CAT* genes, such as *At_CAT3*, *Sl_CatX3*, and *St_CatX3*, were highly expressed in all tissues (Fig. 2, Fig. 6). *At_CAT3*, *Sl_CatX3*, and *St_CatX3* were most highly expressed in the stem, fruit, and stamen, respectively. By contrast, duplicated *CAT* genes, including *At_CAT2*, *Sl_CatX2*, *St_CatX2*, and *Ca_CatX2*, showed high expression in both source and sink organs, such as the flower and stamen. *At_CAT1*, *Sl_CatX1*, *St_CatX1*, and *Ca_CatX1* were highly expressed in certain tissues (such as mature pollen, flower, and stamen), suggesting that these *CAT* genes have functionally differentiated following gene duplication.

## Discussion

### Lineage-specific loss of *CAT* genes in Fabaceae

The gene number of *CAT* genes varied substantially (0–8) among algae and higher plants; however, variation in the gene number of *CAT*s was low within angiosperms (Fig. 1). Only one *CAT* member was detected in all members of Fabaceae, except for soybean, which had four *CAT* copies due to whole-genome duplication [25]. As the retention of multiple copies of the same gene has often been observed in the palaeopolyploid genome of soybean [26], the four *CAT* copies in soybean likely belong to a single gene.

We found that *Medtr1386s0010*, a *CAT* gene of *M. truncatula*, was incorrectly annotated in the NCBI database. Thus, members of Fabaceae likely only possessed one *CAT* member. Phylogenetic analysis revealed that all *CAT* genes from Fabaceae formed a Fabaceae-specific clade within group β2 (Fig. 2). Given that both group α1 and β1 were the oldest clades in plants, the presence of a single *CAT* member in Fabaceae likely reflects a lineage-specific loss of *CAT* members. Gene loss might have a greater impact on organisms than most amino acid substitutions; it is thus one of the major drivers of gene family evolution, organogenesis, morphological diversity, and adaptation [27–29]. The presence of a single *CAT* gene in Fabaceae might be related to the special organogenesis and morphological characteristics of members of this group.

Single-copy genes tend to show higher expression levels and sequence identity in more tissues than non-single-copy genes in a species [28]. Consistent with this expectation, *CAT* sequences of Fabaceae were more similar (94.77%) than those of Solanaceae (87.13%) and Brassicaceae (87.33%) (Table S7).

### Intron acquisition promoted the evolution of multiple *CAT* copies in most angiosperms

Gene duplication is one of the main engines driving evolutionary novelties, as it can often lead to neofunctionalization and subfunctionalization through alterations in expression levels or coding sequences [30, 31]. *CAT* genes underwent independent duplication events following the divergence of monocots and eudicots, which resulted in different evolutionary patterns between the two lineages. Multiple copies in a plant genome may be produced by a single copy of a *CAT* gene [3]. New copies of *CAT* genes were obtained through the loss of introns from intron-rich ancestral *CAT* genes in plants [11].

Our results, coupled with the findings of a previous study [3], indicated that new copies of *CAT* genes in plants can be generated through the acquisition of introns from older genes with fewer introns in the same species (Fig. 2, Fig. S2). This was a general pattern among the most angiosperms examined. Except for Fabaceae, the number of introns of group β2 *CAT* genes (5–7) was always higher than that of group β1 CAT genes (2–6) (Fig. S2). These findings suggest that the evolution of angiosperm *CAT* genes was mainly driven by the acquisition of introns. Prokaryotic *CAT* genes naturally lack introns [3], and some basal bacterial lineages possess intronless *CAT* alleles [32]; both of these findings support our interpretation. In Fabaceae, *Gm_CAT1/cat2* and *Gm_CAT3* possessed six introns, whereas the remaining *CAT* genes contained seven introns. Due to the Fabaceae *CAT* genes lacking in group β1, its evolutionary pattern was uncertain.

The intron phases of some gene families, such as the *PDAT* [33], *rhomboid* [34], and *RNAP* [35] families, are conserved among orthologs in higher plants. Introns have a wide range of functions in contemporary species and are involved in almost every step of mRNA processing [36]. Some highly expressed genes typically have higher intron gain rates [37]. We observed large differences in the intron phase of *CAT* genes within the same subfamily, suggesting that the *CAT* gene may affect gene expression or function between different species.

### Acquisition of *CAT* genes by eukaryotic genomes via LGT

Several DNA fragments containing functional genes can be transferred from bacteria to eukaryotes, and this process is referred to as LGT [38, 39]. Generally, sequences transferred to eukaryotes retain their functionality and acquire eukaryotic characteristics [39]. A previous analysis of the *CAT* gene family has shown that several LGT events have occurred not only between bacteria and fungi but also between bacterial and protist ancestors of the green lineage [3].

Our analysis suggested that *CAT* genes from bacteria or protists were transferred to green lineage taxa by LGT events and led to the formation of two types: α and β (Fig. 3a). α-type *CAT* genes comprised all *CAT* genes from charophytes and part of the *CAT* genes from lower land plants (including *P. patens*, *M. polymorpha*, and *S. moellendorffii*). Previous studies have indicated that typical *CAT* genes comprise three clades: clade 1, 2, and 3 [2, 3]. An ancestral clade 2-type CAT that consists of large subunit catalases (~ 750 residues per subunit) gave rise to small subunit catalases (~ 500 residues per subunit) in clade 1 and 3 [2, 8, 40]. These three main clades of the CAT family are shown in Fig. 3b and were consistent with the results of previous studies. Clade 1 (referred to as β type in our study) was more closely related to clade 2 than to clade 3 (referred to as α type in our study) according to the phylogenetic tree.

Clade 3 contains a small paralog group of CATs that evolved from genes in Chlorophyte [8]. However, our findings indicate that this small group (α group) was actually derived from Charophyte, not Chlorophyte. The paralog group was present in multiple species, including all *CAT* genes in *K. flaccidum* and some of the *CAT* genes from *P. patens*, *M. polymorpha*, and *S. moellendorffii*. Thus, α small paralog group may be selectively retained by certain species. The eukaryotic genomes hold most of the genes of bacteria [41]. The catalases travel frequently laterally between the eukaryotic host and bacteria [3]. In order to adapt to the eukaryotic intracellular environment in eukaryotic host, they may eventually abandon the metabolic process of bacterial genes [41]. In the evolution of the *CAT* gene family, frequent LGT between eukaryotic hosts and bacteria may lead to the formation of two clades of *CAT* genes with different functions in plants.

The retention of clade 3-type *CAT* genes in Charophyte, *M. polymorpha*, *P. patens*, and *S. moellendorffii* possibly facilitated adaptation to semi-aquatic environments; by contrast, the loss of clade 3-type *CAT* genes in higher plants indicates that these genes are no longer beneficial in a completely terrestrial environment. The loss of several *CAT* genes has been documented to promote adaptation in several species [42]. Nevertheless, clade 1, 2, and 3-type *CAT* genes share a common ancestor (Fig. 3b).

## Changes in key amino acid residues alter the functions of *CAT* genes

A recent study has proposed that the functional features of plants can be altered when a crucial amino acid residue of *Arabidopsis* ROG1/CAT3 is changed from Cys-343 to Thr-343 or when an important amino acid residue of CAT2 is converted from Thr-343 to Cys-343 [23]. Similar observations have been made in rice OsCATA and OsCATC, which possess Cys-343 and Thr-343, respectively, and are orthologs of *Arabidopsis* At_CAT3 and At_CAT2, respectively. A previous sequence analysis of ROG1-like proteins revealed that the Cys-343 residue was distributed among 12 plants (including one in *P. patens*, seven in the grass family, and three in Brassicaceae), indicating that ROG1-like proteins are structurally and functionally conserved in plants [23]. However, our analysis showed that Cys-343 was a species-specific residue that was only present in Brassicaceae, monocots and Pp_CatX7 in *P. patens* (Fig. 4a).

Changes in specific amino acid residues in proteins have been shown to induce functional diversification in various enzymes. A pair of homologous but functionally different enzymes, the C-glycosyltransferases CGTa and CGTb, sequentially catalyzes the biosynthesis of (iso)schaftosides in plants [43].The functions of SbCGTb and SbCGTa can be switched through structural analysis and mutagenesis of key amino acids. Based on our findings and the results of previous studies, there might be some key residues in CATs with substantial functional implications when altered that have yet to be discovered.

## Conclusion

A total of 82 *CATs* were identified from 29 species and could be clustered into three groups. The transfer of plant *CAT* genes from bacteria to core chlorophytes and charophytes by LGT led to the independent evolution of two types of *CAT* genes: α and β. The newer *CAT* genes were produced through intron acquisition in higher plants, and the Cys-343 residue was only present in monocots and Brassicaceae. Furthermore, all *CAT* genes show high overall sequence identity that individual *CAT* genes showed developmental stage and organ-specific expression patterns.

## Materials and methods

### Data retrieval and identification of *CAT* homologs in plants

All recognizable *CAT* genes were obtained via three steps. First, a search was conducted on the NCBI using “catalase” as a keyword, and the *Arabidopsis* CAT sequence (GenBank: CAA45564.1) was obtained. Second, the Hidden Markov Model (HMM) profiles of the conserved CAT domains PF00199 and PF06628 in the Pfam database [44] were downloaded and used as query sequences with an e-value threshold of < 10^–5^. Third, the sequences obtained in the second step were used to retrieve homologous proteins; related sequences from the *Ostreococcus lucimarinus* [45], *Micromonas pusilla* [46], *Volvox carteri* [47], *Chlamydomonas reinhardtii* [48], *Dunaliella salina* [49], *Mesostigma viride* [50], *Physcomitrella patens* [51], *Marchantia polymorpha* [52], *Selaginella moellendorffii* [53], *Ananas comosus* [54], *Brachypodium distachyon* [55], *Oryza sativa* [56], *Zea mays* [57], *Sorghum bicolor* [58], *Solanum lycopersicum* [59], *Solanum tuberosum* [60], *Capsella rubella* [61], *Arabidopsis lyrata* [62], *Arabidopsis thaliana* [63], *Glycine max* [64], *Cicer arietinum* [65], *Trifolium pratense* [66], and *Medicago truncatula* [67] genomes were searched using the Phytozome 13 website (https://phytozome-next.jgi.doe.gov/). *Klebsormidium flaccidum* [68], *Gnetum montanum* [69], and *Capsicum annuum* [70] sequences were downloaded from the *Klebosrmidium* genome project (http://www.plantmorphogenesis.bio.titech.ac.jp/~algae_genome_project/klebsormidium/index.html), the Dryad database (https://datadryad.org/search?utf8=%E2%9C%93&q=gnetophytes), and the Sol Genomics Network (https://solgenomics.net/), respectively. *Nicotiana plumbaginifolia* [71] sequences were obtained from the NCBI database. *Aspergillus nidulans* [72], *Saccharomyces cerevisiae* [73], *Escherichia coli* [74], *Bacillus subtilis* [75], *Chara braunii* [76], and *Dictyostelium discoideum* [77] sequences were obtained from the Ensembl Genomes database (https://ensemblgenomes.org/). The protein sequences of *Anthoceros punctatus* [78] were downloaded from the Hornwort genomes (https://www.hornworts.uzh.ch/en.html). The *CAT* sequences of *Azolla_filiculoides* and *Salvinia cucullata* [79] were searched from the FernBase genome database (https://www.fernbase.org/). Finally, all sequences were submitted to the Pfam database to verify the presence of conserved domains.

### Sequence annotation and genome size

Sequences that were too long or short or affected by obvious errors in the genome assembly were reannotated using Softberry (FGENESH-HMM-based gene structure prediction (http://www.softberry.com)) [80]. Genome sizes of *M. pusilla*, *O. lucimarinus*, *D. salina*, *C. reinhardtii*, *V. carteri*, *P. patens*, *M. polymorpha*, and *S. moellendorffii* were obtained using the Phytozome 13 database. The size of the *K. flaccidum* genome was obtained from a previous study [81]. The genome sizes of the remaining plants were obtained from the Published Plante Genomes website (https://plabipd.de/plant_genomes_pa.ep).

### Species tree and gene tree construction

Data for each species from the evolutionary TimeTree of life (http://timetree.org/about) were used to construct species and gene trees [82]. Trees of *CAT* sequences were built using the maximum likelihood (ML) and neighbor-joining (NJ) methods. MEGA5, MEGA-X software and the online tool LIRMM (http://www.phylogeny.fr/index.cgi) were used to construct phylogenetic trees [83, 84]. The newly produced species and gene trees were displayed using the Interactive Tree of Life online tool (https://itol.embl.de/).

### Sequence alignment and tertiary structure prediction

Multiple sequence alignments for *CATs* were conducted using ClustalX and BioEdit to identify key amino acid residues. The online Phyre2 Server (http://www.sbg.bio.ic.ac.uk/phyre2/html/page.cgi?id=index) was used to predict the tertiary structure of CAT proteins [85]. The generated protein models were visualized in cartoon mode using the PyMOL tool.

### Tissue-specific expression of *CAT* genes

Expression data of *CAT* genes in different tissues of *Arabidopsis*, tomato, and potato were downloaded from the Bio-Analytic Resource for Plant Biology website (http://bar.utoronto.ca/). Previously published RNA sequencing (RNA-seq) data [86] were used to analyze the expression profiles of *CAT* genes in pepper (the elite *Capsicum* line 6421). Expression profiles were determined in the following tissues: leaves at 2, 5, 10, 15, 20, 25, 30, 40, and 50 days after emergence; floral buds at seven different stages (0.25, 0.35, 0.5, 0.8, 1.0, 1.2, and 1.7 cm); petals, stamens, and ovaries with stigmas in fully blossomed flowers; and fruits on 10, 15, 20, 25, 30, 35, 40, 45, and 50 days after flowering. All data were normalized (log_2_(FPKM + 1)), and heat maps were built using TBtools [87].

## Supplementary Information

**Additional file 1: Figure S1.** Phylogenetic relationship of CATs in 29 green plants. Synechocystis sp. PCC 6803 as an outgroup.

**Additional file 2: Figure S2.** Exon-intron structure of the CAT gene family among different species. Exon-intron structure of some species are not shown due to lacked related sequences. In order to visually show the results, intron phases of several genes are manually simulated and framed with dashed lines.

**Additional file 3: Figure S3.** The motif arrangement of CATs in 29 green plants.

**Additional file 4: Figure S4.** Amino acid alignment of CATs from 29 green plants. The critical amino acid residues (Thr343/Cys-343) are framed with black line.

**Additional file 5.** 

**Additional file 6: Table S1.** The detail information of CAT genes in each species.

**Additional file 7: Table S2.** The detail information of reannotated CAT proteins.

**Additional file 8: Table S3.** The details imformation of bacteria, protists, fungi and animals

**Additional file 9: Table S4.** The 3D structure predication for three CATs in Arabidopsis.

**Additional file 10: Table S5.** Homology matrix of 43 CAT sequences in angiosperms. 

**Additional file 11: Table S6. **The identity level of CATs in specific higher plants.

**Additional file 12: Table S7.** The sequence identity of CATs in Solanaceae, Brassicaceae and Fabaceae.

## Data Availability

All methods using plant material were carried out in accordance with relevant guidelines and regulations in this paper. The data used and/or analyzed during the current study are obtained from the Phytozome 13 website (https://phytozome-next.jgi.doe.gov/); the Ensembl Genomes database (https://ensemblgenomes.org/); the Softberry (FGENESH-HMM-based gene structure prediction (http://www.softberry.com)); the Published Plante Genomes website (https://plabipd.de/plant_genomes_pa.ep); TimeTree of life (http://timetree.org/about); the Interactive Tree of Life online tool (https://itol.embl.de/); the Phyre2 Server (http://www.sbg.bio.ic.ac.uk/phyre2/html/page.cgi?id=index); and the Bio-Analytic Resource for Plant Biology website (http://bar.utoronto.ca/).

## Abbreviations

CATs: Catalases
ROS: Reactive oxygen species
GNV: Gene number variation
LGT: Lateral gene transfer
MYA: Million years ago
