# Supplementary figures and images for: Evolution and functional diversification of catalase genes in the green lineage
Source: BMC Genomics. 2022 Jun 1;23:411. doi: 10.1186/s12864-022-08621-6 (PMC9158360; doi:10.1186/s12864-022-08621-6)

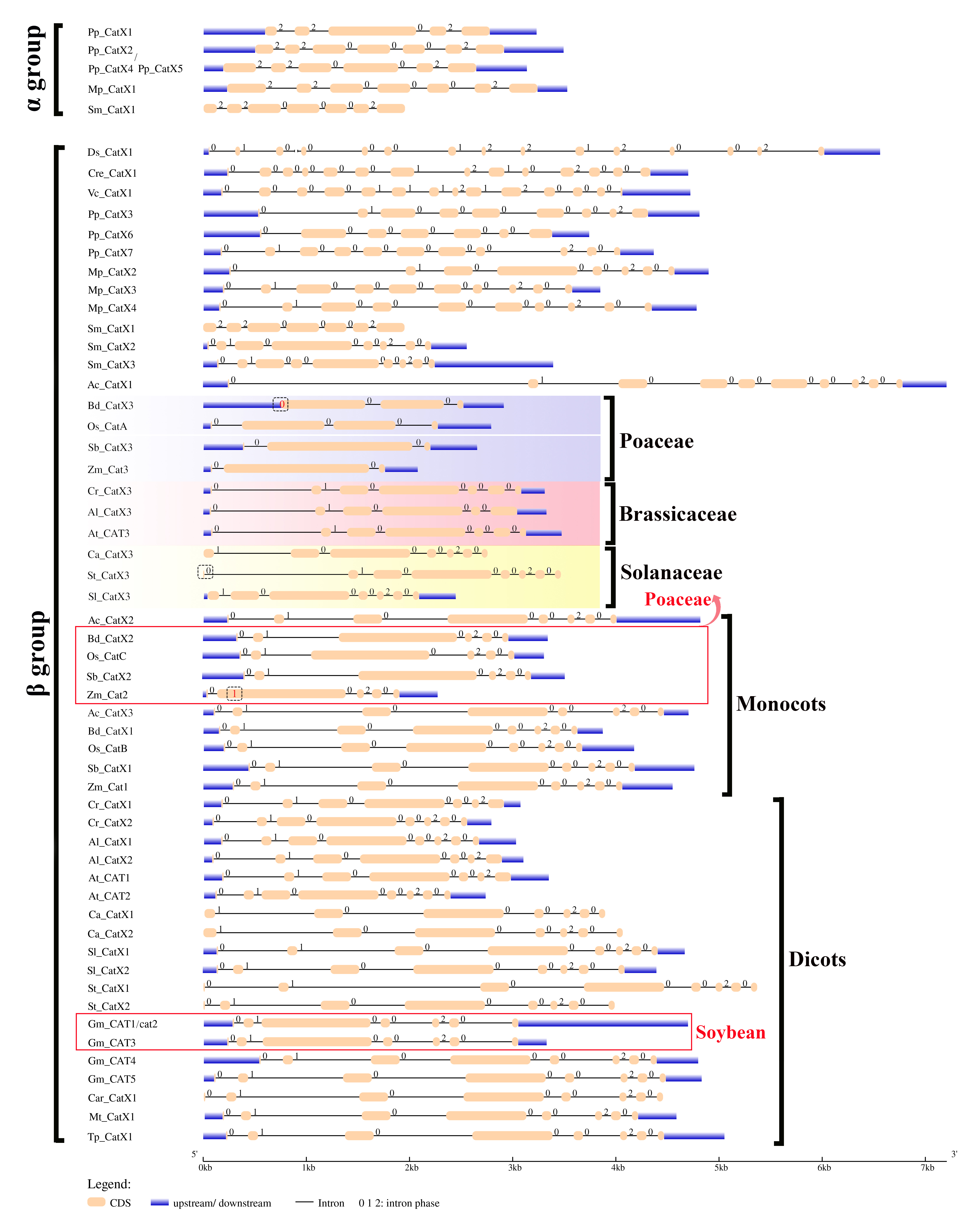

Supplement: Supplementary file 1 — Additional file 1: Figure S1. Phylogenetic relationship of CATs in 29 green plants. Synechocystis sp. PCC 6803 as an outgroup. [file 12864_2022_8621_MOESM1_ESM.jpg]

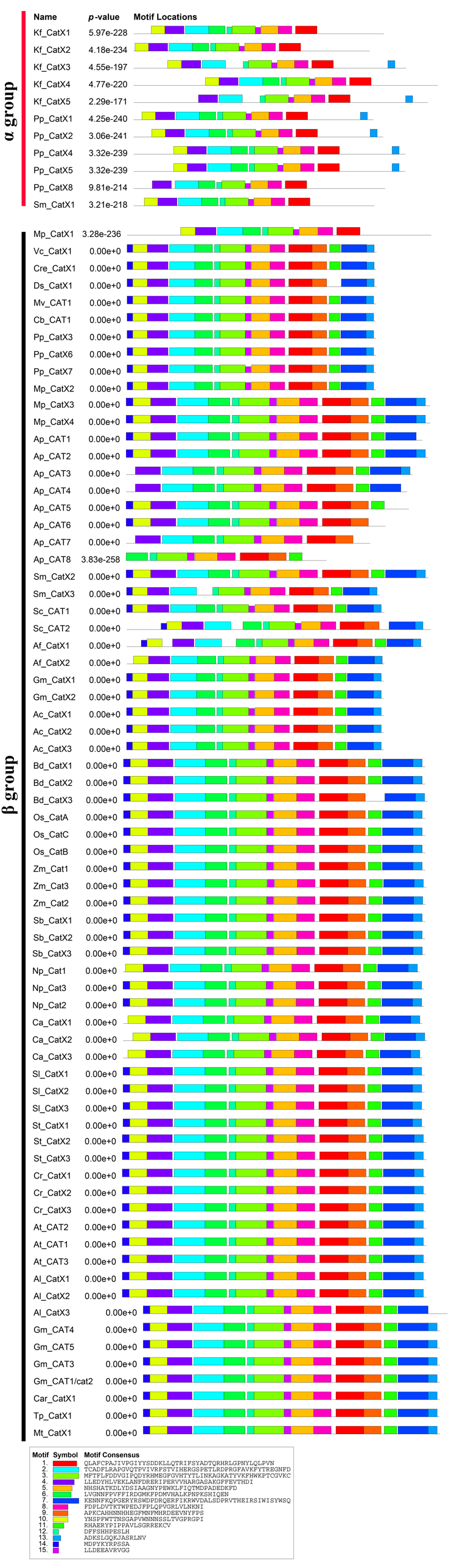

Supplement: Supplementary file 4 — Additional file 4: Figure S4. Amino acid alignment of CATs from 29 green plants. The critical amino acid residues (Thr343/Cys-343) are framed with black line. [file 12864_2022_8621_MOESM4_ESM.jpg]
