# Supplementary material for: Evolution and functional diversification of catalase genes in the green lineage
Source: BMC Genomics. 2022 Jun 1;23:411. doi: 10.1186/s12864-022-08621-6 (PMC9158360; doi:10.1186/s12864-022-08621-6)
Supplement: Supplementary file 5 — Additional file 5. [file 12864_2022_8621_MOESM5_ESM.pdf]

```
        10        20        30        40        50        60        70        80        90       100
....|....|....|....|....|....|....|....|....|....|....|....|....|....|....|....|....|....|....|....|
---------------------------------------------------------------------------------------------------- Vc_CatX1
---------------------------------------------------------------------------------------------------- Cre_CatX1
---------------------------------------------------------------------------------------------------- Ds_CatX1
---------------------------------------------------------------------------------------------------- Mv_CAT1
---------------------------------------------------------------------------------------------------- Cb_CAT1
---------------------------------------------------------------------------------------------------- Os_CatC
---------------------------------------------------------------------------------------------------- Bd_CatX2
---------------------------------------------------------------------------------------------------- Zm_Cat2
---------------------------------------------------------------------------------------------------- Sb_CatX2
---------------------------------------------------------------------------------------------------- Ac_CatX2
---------------------------------------------------------------------------------------------------- Zm_Cat1
---------------------------------------------------------------------------------------------------- Sb_CatX1
---------------------------------------------------------------------------------------------------- Os_CatB
---------------------------------------------------------------------------------------------------- Bd_CatX1
---------------------------------------------------------------------------------------------------- Ac_CatX3
---------------------------------------------------------------------------------------------------- Sl_CatX1
---------------------------------------------------------------------------------------------------- St_CatX1
---------------------------------------------------------------------------------------------------- Np_Cat3
---------------------------------------------------------------------------------------------------- Ca_CatX1
---------------------------------------------------------------------------------------------------- At_CAT1
---------------------------------------------------------------------------------------------------- Al_CatX2
---------------------------------------------------------------------------------------------------- Cr_CatX1
---------------------------------------------------------------------------------------------------- Sl_CatX2
---------------------------------------------------------------------------------------------------- St_CatX2
---------------------------------------------------------------------------------------------------- Ca_CatX2
---------------------------------------------------------------------------------------------------- Np_Cat1
---------------------------------------------------------------------------------------------------- At_CAT2
---------------------------------------------------------------------------------------------------- Al_CatX1
---------------------------------------------------------------------------------------------------- Cr_CatX2
---------------------------------------------------------------------------------------------------- Gm_CAT4
---------------------------------------------------------------------------------------------------- Gm_CAT5
---------------------------------------------------------------------------------------------------- Mt_CatX1
---------------------------------------------------------------------------------------------------- Tp_CatX1
---------------------------------------------------------------------------------------------------- Car_CatX1
---------------------------------------------------------------------------------------------------- Gm_CAT3
---------------------------------------------------------------------------------------------------- Gm_CAT1/cat2
---------------------------------------------------------------------------------------------------- Gm_CatX1
----------------------------------------------------------------------------------MASSVCAASFSRIIHQLS Sc_CAT2
---------------------------------------------------------------------------------------------------- Sm_CatX2
---------------------------------------------------------------------------------------------------- Sm_CatX3
---------------------------------------------------------------------------------------------------- Mp_CatX2
---------------------------------------------------------------------------------------------------- Sc_CAT1
---------------------------------------------------------------------------------------------------- Ap_CAT5
---------------------------------------------------------------------------------------------------- Ap_CAT8
---------------------------------------------------------------------------------------------------- Ap_CAT1
---------------------------------------------------------------------------------------------------- Ap_CAT3
---------------------------------------------------------------------------------------------------- Ap_CAT4
---------------------------------------------------------------------------------------------------- Ap_CAT6
---------------------------------------------------------------------------------------------------- Ap_CAT7
---------------------------------------------------------------------------------------------------- Pp_CatX3
---------------------------------------------------------------------------------------------------- Pp_CatX6
---------------------------------------------------------------------------------------------------- Mp_CatX3
---------------------------------------------------------------------------------------------------- Mp_CatX4
---------------------------------------------------------------------------------------------------- Ap_CAT2
---------------------------------------------------------------------------------------------------- Os_CatA
---------------------------------------------------------------------------------------------------- Bd_CatX3
---------------------------------------------------------------------------------------------------- Zm_Cat3
---------------------------------------------------------------------------------------------------- Sb_CatX3
---------------------------------------------------------------------------------------------------- Ac_CatX1
---------------------------------------------------------------------------------------------------- Sl_CatX3
---------------------------------------------------------------------------------------------------- St_CatX3
---------------------------------------------------------------------------------------------------- Ca_CatX3
---------------------------------------------------------------------------------------------------- Np_Cat2
---------------------------------------------------------------------------------------------------- At_CAT3
---------------------------------------------------------------------------------------------------- Cr_CatX3
---------------------------------------------------------------------------------------------------- Al_CatX3
---------------------------------------------------------------------------------------------------- Pp_CatX7
---------------------------------------------------------------------------------------------------- Gm_CatX2
---------------------------------------------------------------------------------------------------- Pp_CatX8
---------------------------------------------------------------------------------------------------- Kf_CatX1
MLGAWKAGRPNWGSGVPGRKAVGALAALVGLLLVASIVHRLLALPVTYHSAPGDSLRVVELYGQWKNPAERDRGAGLVFVSRALNCTKWVEPTYDQQKTA Kf_CatX4
---------------------------------------------MTFEMATLAPIRG---------LAPRNSCAPLRAH----------ESSDGLRPSL Pp_CatX4
---------------------------------------------MTFEMATLAPIRG---------LAPRNSCAPLRAH----------ESSDGLRPSL Pp_CatX5
---------------------------------------------------------------------------------------------------- Pp_CatX2
---------------------------------------------------------------------------------------------------- Pp_CatX1
----------------------------------------MASYTSVSSMSALSVIGSSNCHPKSKRQPNPGTCSGCSAS----------PISFALKSTF Mp_CatX1
---------------------------------------------------------------------------------------------------- Sm_CatX1
---------------------------------------------------------------------------------------------------- Kf_CatX2
-----------------------------------------MASSAMASRVAEKGLQGQSIFQRVQDNKQFLTGTAFAAP-------------------- Kf_CatX3
---------------------------------------MSASSVRLSLSPCISALRSSSSYAGSAPLRQFGFPAIFSTSG-------------KRYHAT Kf_CatX5
                                                                                   Clustal Consensus
```

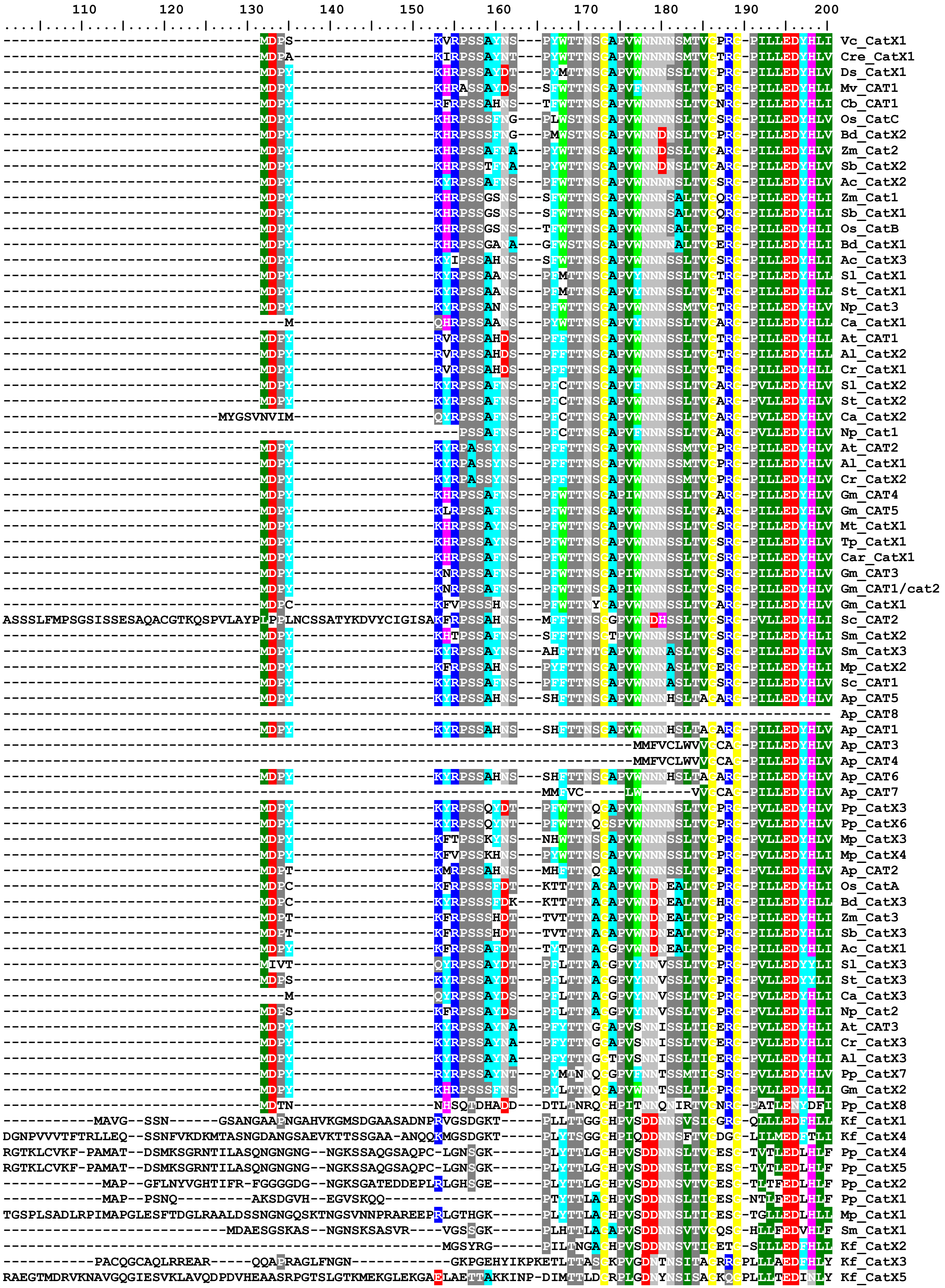
110
120
130
140
150
160
170
180
190
200
Vc_CatX1
Cre_CatX1
Ds_CatX1
Mv_CAT1
Cb_CAT1
Os_CatC
Bd_CatX2
Zm_Cat2
Sb_CatX2
Ac_CatX2
Zm_Cat1
Sb_CatX1
Os_CatB
Bd_CatX1
Ac_CatX3
Sl_CatX1
St_CatX1
Np_Cat3
Ca_CatX1
At_CAT1
Al_CatX2
Cr_CatX1
Sl_CatX2
St_CatX2
Ca_CatX2
Np_Cat1
At_CAT2
Al_CatX1
Cr_CatX2
Gm_CAT4
Gm_CAT5
Mt_CatX1
Tp_CatX1
Car_CatX1
Gm_CAT3
Gm_CAT1/cat2
Gm_CatX1
Sc_CAT2
Sm_CatX2
Sm_CatX3
Mp_CatX2
Sc_CAT1
Ap_CAT5
Ap_CAT8
Ap_CAT1
Ap_CAT3
Ap_CAT4
Ap_CAT6
Ap_CAT7
Pp_CatX3
Pp_CatX6
Mp_CatX3
Mp_CatX4
Ap_CAT2
Os_CatA
Bd_CatX3
Zm_Cat3
Sb_CatX3
Ac_CatX1
Sl_CatX3
St_CatX3
Ca_CatX3
Np_Cat2
At_CAT3
Cr_CatX3
Al_CatX3
Pp_CatX7
Gm_CatX2
Pp_CatX8
Kf_CatX1
Kf_CatX4
Pp_CatX4
Pp_CatX5
Pp_CatX2
Pp_CatX1
Mp_CatX1
Sm_CatX1
Kf_CatX2
Kf_CatX3
Kf_CatX5

Clustal Consensus

```
         210       220       230       240       250       260       270       280       290       300
....|....|....|....|....|....|....|....|....|....|....|....|....|....|....|....|....|....|....|....|
EKLAQFDRERIPERVVHARGAAAKGFFEVT-----HNVSHLTAADFLRAPGVQTPVIVRFSTVVHERGSPETLRDPRGFAVKFYTRE------------- Vc_CatX1
EKLAQFDRERIPERVVHARGAAAKGFFEVT-----HDISALTAADFLRAPGVQTPVIVRFSTVVHERGSPETLRDPRGFAVKFYTRE------------- Cre_CatX1
EKLAQFDRERIPERVVHARGAVAKGFFEVT-----HDVSHLTCADFLRAPGVQTPVIVRFSTVVHERGSPETLRDPRGFAVKFYTRE------------- Ds_CatX1
EKLRNFDSERIPERVVHARGASAKGFFEVT-----HDVSHLTCADFLRAPGVQTPMIVRFSTVIHERGSPETLRDPRGFAVKFYTRE------------- Mv_CAT1
EKLANFDRERIPERVVHARGASAKGFFEVT-----HDVSHLTCADFLRAPGVVTPLIVRFSTVIHERGSPETIRDPRGFAVKFYTRE------------- Cb_CAT1
EKLANFDRERIPERVVHARGASAKGFFEVT-----HDITHLTCADFLRAPGVQTPVIVRFSTVIHERGSPETLRDPRGFAIKFYTRE------------- Os_CatC
EKIANFDRERIPERVVHARGASAKGFFEVT-----HDISHLTCADFLRAPGVQTPVIVRFSTVIHERGSPETLRDPRGFAIKFYTRE------------- Bd_CatX2
EKLANFDRERIPERVVHARGASAKGFFEVT-----HDITHLTCADFLRAPGVQTPVIVRFSTVIHERGSPETLRDPRGFAVKFYTRE------------- Zm_Cat2
EKLANFDRERIPERVVHARGASAKGFFEVT-----HDITHLSCADFLRAPGVQTPVIVRFSTVIHERGSPETLRDPRGFAVKFYTRE------------- Sb_CatX2
EKLAQFDRERIPERVVHARGASAKGFFEVT-----HDISHLTCADFLRAPGVQTPVIVRFSTVIHERGSPETLRDPRGFAVKFYTRE------------- Ac_CatX2
EKLAQFDRERIPERVVHARGASAKGFFEVT-----HDVSHLTCADFLRAPGVQTPVIVRFSTVVHERGSPETLRDPRGFAVKFYTRE------------- Zm_Cat1
EKLAQFDRERIPERVVHARGASAKGFFEVT-----HDISHLTCADFLRAPGVQTPVIVRFSTVVHERGSPETLRDPRGFAVKFYTRE------------- Sb_CatX1
EKLAQFDRERIPERVVHARGASAKGFFEVT-----HDISHLTCADFLRAPGVQTPVIVRFSTVVHERGSPETLRDPRGFAVKFYTRE------------- Os_CatB
EKLAQFDRERIPERVVHARGASAKGFFEVT-----HDISHLTCADFLRAPGVQTPVIVRFSTVVHERGSPETLRDPRGFAVKFYTRE------------- Bd_CatX1
EKLAQFDRERIPERVVHARGASAKGFFEVT-----HDISHLTCADFLRAPGVQTPVIVRFSTVIHERGSPETLRDPRGFAVKFYTRE------------- Ac_CatX3
EKIANFDRERIPERVVHARGASAKGFFEVT-----HDISHLTCADFLRAPGVQTPVIVRFSTVIHERGSPETLRDPRGFAVKFYTRE------------- Sl_CatX1
EKLANFDRERIPERVVHARGASAKGFFEVT-----HDISHLTCADFLRAPGVQTPVIVRFSTVIHERGSPETLRDPRGFAVKFYTRE------------- St_CatX1
EKLANFDRERIPERVVHARGASAKGFFEVT-----HDITHLTCADFLRAPGVQTPVIVRFSTVIHERGSPETLRDPRGFAVKFYTRE------------- Np_Cat3
EKLANFDRERIPERVVHARGASAKGFFEVT-----HDITRLTCADFLRAPGVQTPVIVRFSTVIHERGSPETLRDPRGFAVKFYTRE------------- Ca_CatX1
EKLANFDRERIPERVVHARGASAKGFFEVT-----HDITQLTSADFLRGPGVQTPVIVRFSTVIHERGSPETLRDPRGFAVKFYTRE------------- At_CAT1
EKLANFDRERIPERVVHARGASAKGFFEVT-----HDITQLTSADFLRGPGAQTPVIVRFSTVIHERGSPETLRDPRGFAVKFYTRE------------- Al_CatX2
EKIANFDRERIPERVVHARGASAKGFFEVT-----HDITQLTSADFLRGPGVQTPVIVRFSTVIHERGSPETLRDPRGFAVKFYTRE------------- Cr_CatX1
EKLANFDRERIAERVVHARGASAKGFFEVT-----HDIAHLTCADFLRAPGVQTPVIVRFSTVIHERGSPETLRDPRGFAVKFYTRE------------- Sl_CatX2
EKLANFDRERIAERVVHARGASAKGFFEVT-----HDIAHLTCADFLRAPGVQTPVIVRFSTVIHERGSPETLRDPRGFAVKFYTRE------------- St_CatX2
EKLANFDRERIAERVVHARGASAKGFFEVT-----HDITHLTCADFLRAPGVQTPVIVRFSTVIHERGSPETLRDPRGFAVKFYTRE------------- Ca_CatX2
EKLANFDRERVPERVVHARGASAKGFFEVT-----HDITHLTCADFLRAPGVQTPVIVRFSTVIHERGSPETLRDPRGFAVKFYTRE------------- Np_Cat1
EKLANFDRERIPERVVHARGASAKGFFEVT-----HDISNLTCADFLRAPGVQTPVIVRFSTVIHERGSPETLRDPRGFAVKFYTRE------------- At_CAT2
EKLANFDRERIPERVVHARGASAKGFFEVT-----HDISNLTCADFLRAPGVQTPVIVRFSTVIHERGSPETLRDPRGFAVKFYTRE------------- Al_CatX1
EKLANFDRERIPERVVHARGASAKGFFEVT-----HDISNLTCADFLRAPGVQTPVIVRFSTVIHERGSPETLRDPRGFAVKFYTRE------------- Cr_CatX2
EKLANFDRERIPERVVHARGASAKGFFEVT-----HDISHLTCADFLRAPGVQTPVIVRFSTVIHERGSPETLRDPRGFAVKFYTRE------------- Gm_CAT4
EKLANFDRERIPERVVHARGASAKGFFEVT-----HDISHLTCADFLRAPGVQTPVIVRFSTVIHERGSPETLRDPRGFAVKFYTRE------------- Gm_CAT5
EKLAQFDRERIPERVVHARGASAKGFFEVT-----HDISHLTCADFLRAPGVQTPVIVRFSTVIHERGSPETLRDPRGFAVKFYTRE------------- Mt_CatX1
EKLAQFDRERIPERVVHARGASAKGFFEVT-----HDVSHLTCADFLRAPGVQTPVIVRFSTVIHERGSPETLRDPRGFAVKFYTRE------------- Tp_CatX1
EKLANFDRERIPERVVHARGASAKGFFEVT-----HDVSHLTCADFLRAPGVQTPVIVRFSTVIHERGSPETLRDPRGFAVKFYTRE------------- Car_CatX1
EKLANFDRERIPERVVHARGASAKGFFEVT-----HDISHLTCADFLRAPGVQTPLIVRFSTVIHERGSPETLRDPRGFAVKFYTRE------------- Gm_CAT3
EKLANFDRERIPERVVHARGASAKGFFEVT-----HDISHLTCADFLRAPGVQTPLIVRFSTVIHERGSPETLRDPRGFAVKFYTRE------------- Gm_CAT1/cat2
EKLAQFDRERIPERVVHARGATAKGFFEVT-----HDVSHLTCADFLRAPGVQTPVIVRFSTVVHERGSPETLRDPRGFAVKFYTRE------------- Gm_CatX1
EKLAQFDRERIPERVVHARGATAKGFFEVT-----HDVSHLTCADFLRAPGVQTPLITRFSTVIHERGSPETLRDPRGFAVKFYTREIVSVSPQLQIIGS Sc_CAT2
EKLAQFDRERIPERVVHARGASAKGYFEVT-----HDISNLTCADFLRAPGVQTPLIVRFSTVIHERGSPETLRDPRGFAVKFYTRE------------- Sm_CatX2
EKLAQFDRERIPERVVHARGASAKGFFEVT-----HDVSHLTCADFLRAPGVQTPVIVRFSTVIHERGSPETLRDPRGFAVKFYTRE------------- Sm_CatX3
EKIAQFDRERIPERVVHARGASAKGFFEVT-----HDISHLTCADFLRAPGVQTPLIVRFSTVIHERGSPESIRDPRGFAVKFYTRE------------- Mp_CatX2
EKIAQFDRERIPERVVHARGASAKGFFEVT-----HDVSHLTCADFLRAPGVQTPLIVRFSTVIHERGSPETLRDPRGFAVKFYTRE------------- Sc_CAT1
EKIAQFDRERIPERVVHARGASAKGFFEVT-----HDVSHLTCADFLRAPGVQTPIILRFSTVIHERGSPESLRDPRGFAIKFYTRE------------- Ap_CAT5
---------------------------------------------------------------------------------------------------- Ap_CAT8
EKIAQFDRERIPERVVHARGASAKGFFEVT-----HDVSHLTCADFLRAPGVQTPIILRFSTVIHERGSPESLRDPRGFAIKFYTRE------------- Ap_CAT1
EKIAQFDRERIPERVVHARGASAKGFFEVT-----HDVSHLTCADFLRAPGVQTPIILRFSTVIHERGSPESLRDPRGFAIKFYTRE------------- Ap_CAT3
EKIAQFDRERIPERVVHARGASAKGFFEVT-----HDVSHLTCADFLRAPGVQTPIILRFSTVIHERGSPESLRDPRGFAIKFYTRE------------- Ap_CAT4
EKIAQFDRERIPERVVHARGASAKGFFEVT-----HDVSHLTCADFLRAPGVQTPIILRFSTVIHERGSPESLRDPRGFAIKFYTRE------------- Ap_CAT6
EKIAQFDRERIPERVVHARGASAKGFFEVT-----HDVSHLTCADFLRAPGVQTPIILRFSTVIHERGSPESLRDPRGFAIKFYTRE------------- Ap_CAT7
EKLAQFDRERIPERVVHARGASAKGFFEVT-----HDISHLTCADFLRAPGVQTPVIVRFSTVIHERGSPETLRDPRGFAVKFYTRE------------- Pp_CatX3
EKLAQFDRERIPERVVHARGASAKGFFEVT-----HDISHLTCADFLRAPGVQTPVIVRFSTVIHERGSPEAIRDPRGFAVKFYTRE------------- Pp_CatX6
EKLAQFDRERIPERVVHARGFSAKGYFEVT-----HDISHLTTADFLRAPGVQTPLITRFSTVIHEIGSPETLRDPRGFAVKFYTRE------------- Mp_CatX3
EKLANFDRERIPERVVHSRGFSAKGYFEVT-----HDISHLTCADFLRAPGVQTPLITRFSTVIHERGSPETIRDPRGFAVKFYTRE------------- Mp_CatX4
EKLAQFDRERIPERVVHARGFSAKGFFEVT-----HDVSHLTCADFLRATGVQTPLIVRFSTVIHERGSPETLRDPRGFAVKFYTGE------------- Ap_CAT2
EKVAHFARERIPERVVHARGASAKGFFECT-----HDVTDITCADFLRSPGAQTPVIVRFSTVIHERGSPETIRDPRGFAVKFYTRE------------- Os_CatA
EKVAHFARERIPERVVHARGASAKGFFECT-----HDVTDLTCADFLRAPGARTPVIVRFSTVIHERGSPETIRDPRGFAVKFYTRE------------- Bd_CatX3
EKVAHFARERIPERVVHARGASAKGFFECT-----HDVTSLTCADFLRAPGVRTPVIVRFSTVIHERGSPETIRDPRGFAVKFYTRE------------- Zm_Cat3
EKVAHFARERIPERVVHARGASAKGFFECT-----HDVASLTCADFLRSPGVRTPVIVRFSTVIHERGSPETIRDPRGFAVKFYTRE------------- Sb_CatX3
EKIAHFARERIPERVVHARGASAKGFFECT-----HDVTGLTCADFLRAPGVQTPVIVRFSTVIHERGSPETIRDPRGFAVKFYTRE------------- Ac_CatX1
EKLATFDREKIPERVVHARGASAKGFFEVT-----HDISHLTCADFLRAPGAQTPVICRFSTVVHERGSPESIRDIRGFAVKFYTRE------------- Sl_CatX3
EKLATFDREKIPERVVHARGASAKGFFEVT-----HDISHLSCADFLRAPGAQTPVICRFSTVVHERGSPESIRDIRGFAVKFYTRE------------- St_CatX3
EKLATFDRERIPERVVHARGASAKGFFEVT-----HDISHLTCADFLRAPGVQTPVICRFSTVVHERGSPESIRDIRGFAVKFYTRE------------- Ca_CatX3
EKLATFDRERIPERVVHARGASAKGFFEVT-----HDISHLTCADFLRAPGVQTPVIVRFSTVVHERGSPESLRDIRGFAVKFYTRE------------- Np_Cat2
EKVANFTRERIPERVVHARGISAKGFFEVT-----HDISNLTCADFLRAPGVQTPVIVRFSTVVHERASPETMRDIRGFAVKFYTRE------------- At_CAT3
EKVANFTRERIPERVVHARGISAKGFFEVT-----HDISNLTCADFLRAPGVQTPVIVRFSTVVHERASPETMRDIRGFAVKFYTRE------------- Cr_CatX3
EKVANFTRERIPERVVHARGISAKGFFEVT-----HDISNLTCADFLRAPGVQTPVIVRFSTVVHERASPETMRDIRGFAVKFYTRE------------- Al_CatX3
EKIANFHRERIPERIVHARGAGAKGYFEVT-----HDITHLTCADFLRGVGVQTPLIARFSTVIHEKGSPETLPDPRGFAVKMYTRE------------- Pp_CatX7
EKFATFDREKIPERVVHARGASAKGFFEVT-----HDVSGLTCADFLRAPGVQTPLIVRFSTVIHERGSPETLRDPRGFAVKFYTRE------------- Gm_CatX2
EKISHFDREKVPERIVHARGAGAHGYFEAYGTVGDEPVSKYTRAKLFQEKGKQTPVFVRFSTVVHGLHSPETLRDPRGFAVKFYTED------------- Pp_CatX8
DKLAHFDRERIPERVVHANGSGAHGYFEVT-----QDVSDLCKAAFLSEVGKRIPVLSRFSTVGLESGSADTVRDPRGFSVKFYTEE------------- Kf_CatX1
EKLAHFNRERIPERVVHASGQGGHGYFEVT-----KDVTHLCKANFLSEVGKRTPILARFSTVGFESGGADTVRDPRGFSVKFYTEE------------- Kf_CatX4
EKNAHFNRERVPERVVHAKGAGAHGYFEVT-----KDVSDLCKAGLFSEVGKRTPVFARFSTVTGESGYADTARDPRGFSLKFYTEE------------- Pp_CatX4
EKNAHFNRERVPERVVHAKGAGAHGYFEVT-----KDVSDLCKAGLFSEVGKRTPVFARFSTVTGESGYADTARDPRGFSLKFYTEE------------- Pp_CatX5
EKNAHFNRERIPERVVHAKGAGAHGYFEVT-----HDVTDLCKAKLFDEVGKKTPVFARFSTVTGESGFPDTARDPRGFALKFYTEE------------- Pp_CatX2
EKNAHFNRERIPERVVHAKGAGAHGYFEVT-----HDVTDICKAAFLSEVGKRTPVIGRWSTVTGESGFPDTARDPRGFSIKFYTEE------------- Pp_CatX1
EKIAHFNRERVPERVVHAKGHGAHGYFEVT-----KDVSDLCKAKLFSEVGKRTPIFARFSSVTGESGFVDTNRDPRGFSVKFYTEE------------- Mp_CatX1
EKQARLNRERVPERFVHAKGAGAHGYFEVT-----KDVSNLCKAKFLSEPGKRTPVFARFSSVAAERGFTDLARDVRGFAVKFYTEE------------- Sm_CatX1
DKLAHFDRERIPERVVHAKGAGAHGYFEVT-----HDVSDICKAKFLDRVGKRTRCFTRFSTVGGESGSADTARDPRGFAVKFYTEE------------- Kf_CatX2
EKNVHFNRERIPPRSVHEKGAGAFGTFTVT-----KDISKYTKAKLFDRIGKKTEIFARWSVVAGEHGSADSVRDPRGFAVKFYTDE------------- Kf_CatX3
```

```
EKNAALNRERVVPRVVHAKGVGAFGKFTVTN----SDMAKYTEAKVFE-SGKSTEIFCRWSGVTAEHGNSDSVRDIRGFALKFYTED------------- Kf_CatX5
                                                                                        Clustal Consensus

          310       320       330       340       350       360       370       380       390       400
....|....|....|....|....|....|....|....|....|....|....|....|....|....|....|....|....|....|....|....|
----------GNYDMVGNNMPVFFIRDGMKFPDMVHAFKPNPKSHIQEAWRIMDFLSHHPESAHMLTFLLDDAGIPLNYRHMPGFGVHTFKLINRAGKET Vc_CatX1
----------GNFDMVGNNMPVFFIRDGMKFPDMVHAFKPNPKSHIQEAWRIMDFLSHHPESCHMLTFLLDDAGIPLNYRHMPGFGVHTMKLINKAGRET Cre_CatX1
----------GNFDMVGNNMPVFFVRDGMKFPDMVHAFKPNPKTNQQEPWRIMDFLSHHPESTHMLTFLLDDVGIPKCYRTMPGFGVHAFKMLSRDGRET Ds_CatX1
----------GNFDLVGNNLPVFFIRDGIKFPDMVHALKPNPQTHIQEDWRIWDFFSHVPEAIHMFSFLLDDNGIPSDYRHMEGFGVHTFRLLNKAGKET Mv_CAT1
----------GNFDMVGNNMPVFFIRDGMKFPDMVHALKPNPKNHIQEDWRIMDFFSHHPESLHMFTFLFDDVGIPKNYRHMPGFGVHTFKLINREGKET Cb_CAT1
----------GNWDLVGNNFPVFFIRDGMKFPDMVHSLKPNPKSHVQENWRILDFFSHHPESLHMFTFLFDDIGIPADYRHMDGSGVNTYTLVNRAGKSH Os_CatC
----------GNWDLVGNNFPVFFIRDGMKFPDMVHSLKPNPKSHIQENWRILDFFSHHPESLHMFTFLFDDIGIPADYRHMDGSGVNTYTLVNRAGKSH Bd_CatX2
----------GNWDLVGNNFPVFFIRDGIKFPDMVHALKPNPRTHIQDNWRILDFFSHHPESLHMFSFLFDDVGIPADYRHMDGSGVHTYTLVSRAGTVT Zm_Cat2
----------GNWDLVGNNFPVFFIRDGMKFPDMVHSLKPNPKTHIQENWRILDFFSHHPESLHMFTFLFDDVGIPADYRHMDGSGVHTYTLINRAGKAT Sb_CatX2
----------GNFDLVGNNFPVFFIRDGMKFPDMVHALKPNPKSHIQENWRIVDFFSHHPESLHMFTFLFDDIGIPTDYRHMDGSGVNTYTLINKEGKAH Ac_CatX2
----------GNFDLVGNNMPVFFIRDGMKFPDMVHAFKPNPKTNLQENWRIVDFFSHHPESLHMFTFLFDDVGIPLNYRHMEGFGVNTYTLINRDGKPH Zm_Cat1
----------GNFDLVGNNMPVFFIRDGMKFPDMVHAFKPNPKTNLQENWRIVDFFSHHPESLHMFTFLFDDVGIPLNYRHMEGFGVNTYTLINRDGKPH Sb_CatX1
----------GNFDLVGNNMPVFFIRDGMKFPDMVHAFKPSPKTNMQENWRIVDFFSHHPESLHMFSFLFDDVGIPLNYRHMEGFGVNTYTLINKDGKPH Os_CatB
----------GNFDLVGNNMPVFFIRDGMKFPDMVHAFKPSPKTNMQENWRIVDFFSHHPESLHMFSFLFDDVGIPLNYRHMEGFGVNTYTLINKDGKVH Bd_CatX1
----------GNFDLVGNNFPVFFVRDGMKFPDMVHALKPNPKSHIQENWRIVDFFSHHPESLHMFAFLFDDVGVPLNYRHMDGFGVNTYTLLSKDGKAH Ac_CatX3
----------GNFDLVGNNFPVFFVRDGMKFPDMVHALKPNPKSHIQENWRILDFFSHHPESLHMFSFLFDDLGIPQDYRHMDGFGVNTYMLINKAGKAQ Sl_CatX1
----------GNFDLVGNNFPVFFVRDGMKFPDMVHALKPNPKSHIQENWRILDFFSHHPESLHMFSFLFDDLGIPQDYRHMDGFGVNTYMLINKAGKAQ St_CatX1
----------GNFDLVGNNFPVFFVRDGMKFPDMVHALKPNPKSHIQENWRILDFFSHHPESLHMFSFLFDDLGVPQDYRHMEGSGVNTYMLINKAGKAH Np_Cat3
----------GNFDLVGNNFPVFFVRDGMKFPDMVHALKPNPKSHIQENWRILDFFSHHPESLHMFSFLFDDLGIPQDYRHMEGSGVNTYTLINKAGKAH Ca_CatX1
----------GNFDLVGNNFPVFFVRDGMKFPDMVHALKPNPKSHIQENWRILDFFSHHPESLHMFSFLFDDLGIPQDYRHMEGAGVNTYMLINKAGKAH At_CAT1
----------GNFDLVGNNFPVFFVRDGMKFPDMVHALKPNPKSHIQENWRILDFFSHHPESLHMFSFLFDDLGIPQDYRHMEGAGVNTYMLINKAGKAH Al_CatX2
----------GNFDLVGNNFPVFFIRDGMKFPDMVHALKPNPKSHIQENWRILDFFSHHPESLHMFSFLFDDLGIPQDYRHMEGAGVNTYMLINKAGKAH Cr_CatX1
----------GNFDLVGNNFPVFFIRDGMKFPDMVHALKPNPKSHIQENWRVLDFFSHHPESLHMFTFLFDDIGIPQDYRHMDGSGVHTFTLINRAGKST Sl_CatX2
----------GNFDLVGNNFPVFFIRDGMKFPDMVHALKPNPKSHIQENWRVLDFFSHHPESLHMFTFLFDDIGIPQDYRHMDGSGVHTFTLINRAGKST St_CatX2
----------GNFDLVGNNFPVFFIRDGMKFPDMVHSLKPNPKSHIQENWRVLDFFSHHPESLHMFTFLFDDIGIPQDYRHMDGSGVHTFTLINKAGKST Ca_CatX2
----------GNFDLVGNNFPVFFIRDGMKFPDMVHALKPNPKSHIQENWRVLDFFSHVPESLHMFTFLFDDIGIPQDYRHMDGSGVHTFTLINKAGKST Np_Cat1
----------GNFDLVGNNFPVFFIRDGMKFPDMVHALKPNPKSHIQENWRILDFFSHHPESLNMFTFLFDDIGIPQDYRHMDGSGVNTYMLINKAGKAH At_CAT2
----------GNFDLVGNNFPVFFIRDGMKFPDMVHALKPNPKSHIQENWRILDFFSHHPESLNMFTFLFDDIGIPQDYRHMDGSGVNTYMLINKAGKAH Al_CatX1
----------GNFDLVGNNMPVFFIRDGMKFPDMVHALKPNPKSHIQENWRILDFFSHHPESLNMFTFLFDDIGIPQDYRHMDGSGVNTYMLINKAGKAH Cr_CatX2
----------GNFDLVGNNLPVFFVRDGMKFPDMVHALKPNPKNHIQENWRILDFFSHFPESLHMFTFLFDDLGVPQDYRHMDGFGVNTYTLINKAGKAV Gm_CAT4
----------GNFDLVGNNLPVFFVRDGMKFPDMVHALKPNPKNHIQENWRILDFFSHFPESLHMFTFLFDDLGVPQDYRHMDGFGVNTYTLINKAGKAV Gm_CAT5
----------GNFDLVGNNFPVFFVHDGMNFPDMVHALKPNPKSHIQENWRILDFFSHFPESLHMFSFLFDDVGVPQDYRHMDGFGVNTYSLINKAGKAV Mt_CatX1
----------GNFDLTGNNFPVFFVHDGMNFPDMVHALKPNPKSHIQENWRILDFFSHFPESLHMFSFLFDDLGVPQDYRHMDGFGVNTYSLINKAGKQL Tp_CatX1
----------GNFDLVGNNFPVFFVHDGMKFPDMVHALKPNPKSHIQENWRILDFFSHFPESLHMFSFLFDDVGVPQDYRHMDGFGVNTYTLINKAGKAL Car_CatX1
----------GNFDLVGNNFPVFFVRDGLKFPDMVHALKPNPKSHIQENWRILDFFSHHPESLHMFSFLFDDVGIPQDYRHMDGFGVNTYTLINKAGKAV Gm_CAT3
----------GNFDLVGNNFPVFFVRDGLKFPDMVHALKPNPKSHIQENWRILDFFSHHPESLHMFSFLFDDVGIPQDYRHMDGFGVNTYTLINKAGKAL Gm_CAT1/cat2
----------GNFDLVGNNFPVFFIRDGIKFPDMVHAFKPNPKTHIQENWRILDFFSHHPESLHMFSFLFDDMGIPLNYRHMEGSGVHTFTFLNKAGKRT Gm_CatX1
RSAVLNCILQGNFDLVGNNFPVFFVRDGIKFPDVIHALKPNPKTHIQEKWRIMDFFSHHPESLNMFSFLFDDLGIPLNYRHMDGSGVNTYSLINKEGKAT Sc_CAT2
----------GNFDLVGNNIPVFFVRDGIKFPDMIHALKPNPKTHVQEPWRIMDHFSHHPESCHMFTFLFDDHGIPLNYRHMEGFGVHTFTLVNRAGKCT Sm_CatX2
----------GNFDMVGNNFPVFFVRDGMKFPDMVHALKPNPRTHIQ--------FSHHPESLHMFTFLLDDIGIPLNYRHMEGFGVHTFTLVNRAGKCS Sm_CatX3
----------GNFDMVGNNMPVFFVRDGMKFPDMVHALKPNPKNHIQEIWRILDFFSHHPESLHMFTFLFDDVGIPLNYRHMEGFGVHTFTLVNKAGKAT Mp_CatX2
----------GNFDMVGNNFPVFFVRDGMKFPDMVHALKPNPKSHIQEEWRILDFFSHHPESLHMFTFLMDDVGIPLNYRHMEGFGVHTFTLMNKAGKAT Sc_CAT1
----------GNFDMVGNNMPVFFVRDGMKFPDMVHALKPNPKNHIQEIWRILDFFSHHPESCHMFTFLFDDVGIPKDYRHMPGFGVHTFSLINRVGKQT Ap_CAT5
--------------MVGNNMPVFFVRDGMKFPDMVHALKPNPKNHIQEIWRILDFFSHHPESCHMFTFLFDDVGIPKDYRHMPGFGVHTFSLINRVGKQT Ap_CAT8
----------GNFDMVGNNMPVFFVRDGMKFPDMVHALKPNPKNHIQEIWRILDFFSHHPESCHMFTFLFDDVGIPKDYRHMPGFGVHTFSLINRVGKQT Ap_CAT1
----------GNFDMVGNNMPVFFVRDGMKFPDMVHALKPNPKNHIQEIWRILDFFSHHPESCHMFTFLFDDVGIPKDYRHMPGFGVHTFSLINRVGKQT Ap_CAT3
----------GNFDMVGNNMPVFFVRDGMKFPDMVHALKPNPKNHIQEIWRILDFFSHHPESCHMFTFLFDDVGIPKDYRHMPGFGVHTFSLINRVGKQT Ap_CAT4
----------GNFDMVGNNMPVFFVRDGMKFPDMVHALKPNPKNHIQEIWRILDFFSHHPESCHMFTFLFDDVGIPKDYRHMPGFGVHTFSLINRVGKQT Ap_CAT6
----------GNFDMVGNNMPVFFVRDGMKFPDMVHALKPNPKNHIQEIWRILDFFSHHPESCHMFTFLFDDVGIPKDYRHMPGFGVHTFSLINRVGKQT Ap_CAT7
----------GNFDMVGNNIPVFFIRDGIKFPDMIHALKPNPKSHIQEPWRIMDFFSHVPEACNMFTFVFDDIGIPADYRHMEGFGVHTFKLITKAGKET Pp_CatX3
----------GNFDMVGNNIPVFFIRDGIKFPDMIHALKPNPKSHIQEAWRIMDFFSHVPESCNMFTFLFDDIGIPADYRHMEGFGVHTFKLINKAGKET Pp_CatX6
----------GNFDMVGNNIPVFFIRDGMKFPDMIHSLKPNPKTNVQEMWRAMDFFSHIPESLHMFSFLFDDWGVPYNFRHMEGFGVHTFKLINKAGKET Mp_CatX3
----------GNFDIVGNNTPVFFIRDAIQFPDVIHAFKPNPKTNIQEYWRVMDFLSHFPESLHTFMFLFDDWGIPSDYRHMEGFGVHTYKLINKAGKET Mp_CatX4
----------GNFDMVGNNIPVFFIRDGMQFPDMIHALKPNPRTHIQESWRMMDYFSFVPESCHMFTFLFDDWGIPADFRHMEGFGVHTFKLINREGKET Ap_CAT2
----------GNWDLLGNNFPVFFIRDGIKFPDVIHAFKPNPRSHVQEYWRVFDFLSHHPESLHTFFFLFDDVGIPTDYRHMDGFGVNTYTFVTRDAKAR Os_CatA
----------GNWDLLGNNFPVFFIRDGIKFPDVIHAFKPNPRSHVQEYWRVFDFLSHLPESLHTFFFLFDDVGIPTDYRHMEGFGVNTYTFVTREGKSK Bd_CatX3
----------GNWDLLGNNFPVFFIRDGIKFPDVIHAFKPNPRSHVQEYWRVFDFLSHLPESLHTFFFLFDDVGVPSDYRHMEGFGVNTYTFVSAAGKAQ Zm_Cat3
----------GNWDLLGNNFPVFFIRDGIKFPDVIHAFKPNPRSHVQEYWRVFDFLSHLPESLHTFFFLFDDVGVPTDYRHMEGFGVNTYTFVNAAGKSH Sb_CatX3
----------GNWDLLGNNFPVFFIRDGIKFPDVIHAFKPNPKSHIQEYWRVFDFLSHHPESLHTFCFLFDDVGVPTDYRHMEGFGVNTYTFINRAGKAH Ac_CatX1
----------GNFDLVGNNVPVFFNRDAKSFPDTIRALKPNPKSHIQENWRILDFFSFLPESLHTFAFFYDDVCLPTDYRHMEGFGVHAYQLINKEGKAH Sl_CatX3
----------GNFDLVGNNVPVFFNRDAKSFPDTIRALKPNPKSHIQENWRILDFFSFLPESLHTFAFFYDDVCLPTDYRHMEGFGVHAYQLINKEGKAH St_CatX3
----------GNFDLVGNNVPVFFNRDAKSFPDTIRALKPNPKSHIQENWRILDFFSFLPESLHTFAFFYDDVCLPTDYRHMEGFGVHAYQLINKAGKAH Ca_CatX3
----------GNFDLVGNNVPVFFNRDAKSFPDTIRALKPNPKSHIQEYWRILDFFSFLPESLHTFAWFFDDVCLPINYRHMEGYGVHAYQLINKAGKAH Np_Cat2
----------GNFDLVGNNTPVFFIRDGIQFPDVVHALKPNPKTNIQEYWRILDYMSHLPESLLTWCWMFDDVGIPQDYRHMEGFGVHTYTLIAKSGKVL At_CAT3
----------GNFDLVGNNTPVFFIRDGIQFPDVVHALKPNPKTNIQEYWRILDYMSHLPESLLTWTWMFDDVGIPQDYRHMEGFGVHTYTLVAKSGKVL Cr_CatX3
----------GNFDLVGNNTPVFFIRDGIQFPDVVHALKPNPKTNIQEYWRILDYMSHLPESLLTWCWMFDDVGIPQDYRHMEGFGVHTYTLVAKSGKVL Al_CatX3
----------GNFDMVGNNMPVFFVRDAMQFPDVIHAMKPNPKSNVQEWWRIMDYLSYIPESTHMFFWLDDWGVPTDYRHMDGYTVHTFKLINKEGKET Pp_CatX7
----------GNFDIVGNNIPVFFTRDAMQFPDVIRALKPNPKSHIQEPWRYLDFMSHLPESVLTFLYWFDDWGIPTDYRHMDGYGVHTFKLINKAGKET Gm_CatX2
----------GNWDLVGNNLKIFFIRDAMKFPDMIHAFRPDPVTNIQDPRRFFDFCANSPESFHMVTFVYSPWGIPANYRMMQGSGVNTYKWVNNEGKGV Pp_CatX8
----------GIWDMVGNNTPIFFMRDPIKFPDFIHTQKRHPSKHVKDPDVFWDFLTLTPESFHQVLILFSPRGVPDGYRHMNGYGSHTFKWVNKEGQAN Kf_CatX1
----------GNWDMVGNNTPVFFIRDPSKFPDLIHTQKRHPAKHTKDPDMFWDFLSLVPESFHQVLILFSPRGVPVSPRYMNGYGSHTFKWVNKEGQFN Kf_CatX4
----------GNWDMVGNNTPVFFVRDAAKFPDLIHSQKRNPQTHMKDPNAFWDFLSLVPESLHQVLITFSPRGVPDGHRHMHGYGSHTFKWVNKEGKAH Pp_CatX4
----------GNWDMVGNNTPVFFVRDAAKFPDLIHSQKRNPQTHMKDPNAFWDFLSLVPESLHQVLITFSPRGVPDGHRHMHGYGSHTFKWVNKEGKAH Pp_CatX5
----------GNWDMVGNNTPVFFVRDALKFPDLIHSQKRNPQTHMKDPNAFWDFLSLVPESLHQVLITFSPRGVPDGHRHMHGYGSHTYKWINKEGKAH Pp_CatX2
----------GNWDMVGNNTPVFFVRDAIKFPDLIHSQKRNPATHMKDPNAFWDFLSLVPESLHQVLITFSPRGVPDGHRHMHGYGSHTYKWVNKEGKAH Pp_CatX1
----------GNWDMVGNNTPVFFVRDAIKFPDLIHSQKRNPQTHMKDPNAFWDFLSLVPESLHQVLITFSPRGVPDGFRHMHGYGSHTFKWVNSEGKAN Mp_CatX1
----------GNWDMTGNNTPVFWVRDPLKFPDFVHSQKRNPKTNAKDPNAFWDFLSLVPESMHQLLITFSPRGVPATYRNMNGYGSHTFKWVNKERKAH Sm_CatX1
----------GIYDLVGNNTPVFFIRDPLKFPDFIHTQKRNPETHLKDPDMFWDFLSLVPESVHQVMILFSPRGTPDGYRFMNGYGSHTFKWVNDHGKAH Kf_CatX2
```

```
----------GNWDLVGNNTPIFFIRDPLKFPDLIHSQKRDV-RGLRSWTMRWDFWGHSPEATHQVLWLMGDRGLPKSYRHMNGYGSHTFSLVNDNNERV Kf_CatX3
----------GNWDLVGNNSPIFFFRDPVKFPDLVHSLMRDP-RGLKSWVHRWDFWGLTPESVHQVLWLFGDRGIPKSYRYMNGYGVHAFSMINKDKERV Kf_CatX5
          : ***  :*: :*  .*** :::        :         **:        .    *   * * *   ::   :        Clustal Consensus

                 410       420       430       440       450       460       470       480       490       500
....|....|....|....|....|....|....|....|....|....|....|....|....|....|....|....|....|....|....|....|
YVKFHWIPKCGEKYLL-DDEAVLVGGANHSHATKDLYDAIAAGDYPEWTLMIQTMDPADEDKFD-FDPLDVTKTWPESLFPLQPVGRMVLNRNVDNFFND Vc_CatX1
YVKFHWVPKCGEKYLL-DDEAVMVGGANHSHATKDLFDAIAAGDYPEWALMVQTMDPADEDKFD-FDPLDVTKTWPESLFPMQPVGRMVLNRNVDNFFNE Cre_CatX1
FVKFHWKPKQGEHNLL-DDEAVIVGGSNHSHATKDLYEAIAAGDYPEWTLYIQTMDPKDEDKFN-FDPLDVAKTWPEDIFPLQPIGRMVLNRNPDNFFAE Ds_CatX1
LCKFHWVPTCGVKFLM-DDEAVLVGGKNHSHATKDLTDAIARGEYPEWKIMIQTMEPADQDKFD-FDPFDVTKIWPEALFPLQPVGRMVLNKNIDNFHLE Mv_CAT1
YCKFHWVPTCGEECLL-EEEAVVVGGTNHSHATKDLYDAIAGGDYPEWKLYVQTMEIADQFNFD-FDPLDVTKTWPEEMFPLQPVGRMVLNKNIDNFFAE Cb_CAT1
YVKFHWKPTCGVKSLL-DDEAVTVGGTNHSHATQDLYDSIAAGNFPEWKLFIQTIDPDHEDRFD-FDPLDVTKTWPEDIVPLQPVGRMVLNRNIDNFFSE Os_CatC
YVKFHWKPTCGVKSLL-DDEAVTVGGTNHSHATKDLYDSIAAGNYPEWKFYIQIIDPDHEGRFD-FDPLDVTKTWPEDIIPLQPVGRLVLNRNIDNFFSE Bd_CatX2
YVKFHWRPTCGVRSLM-DDEAVAVGGANHSHATKDLTDAIAAGNFPEWTLYIQTMDPEMEDRLDDLDPLDVTKTWPEDAFPLQPVGRLVLNRNIDNFFAE Zm_Cat2
YVKFHWRPTCGVRSLL-DDEAVAVGGANHSHATKDLTDAIAAGNFPEWTLYIQTMDPEHEDRFD-FDPLDVTKTWPEDVFPLQPVGRMVLNRNIDNFFAE Sb_CatX2
YVKFHWKPTCGVKSLL-DDEAVIVGGTNHSHATKDLYDSIAAGNYPEWKLYIQIIDPDYEDRFD-FDPLDVTKTWPEDILPLQPVGRLVLNRNIDNFFAE Ac_CatX2
LVKFHWKPTCGVKCLL-DNEAVTVGGTCHSHATKDLYDSIAAGNYPEWKLYIQTIDLDHEDKFD-FDPLDVTKTWPEDIIPLQPVGRMVLNKNVDNFFAE Zm_Cat1
LVKFHWKPTCGVKCLL-DDEAVTVGGTCHSHATKDLYDSIAAGNYPEWKLYIQTIDLDHEDKFD-FDPLDVTKTWPEDIIPLQPVGRMVLNKNIDNFFAE Sb_CatX1
LVKFHWKPTCGVKCLL-DDEAVTVGGTCHSHATKDLTDSIAAGNYPEWKLYIQTIDPDHEDRFD-FDPLDVTKTWPEDIIPLQPVGRMVLNKNIDNFFAE Os_CatB
LVKFHWKPTCGVKCLL-DDEAVTVGGTCHTHATKDLTDSIAAGNYPEWKLFIQTIDADHEDKFD-FDPLDVTKTWPEDIIPLQPVGRMILNKNIDNFFAE Bd_CatX1
YVKFHWKPTCGVKCLL-EDEAITVGGSNHSHATKDLYDSIQAGNYPEWKLFIQTIDPDHEDRFD-FDPLDVTKTWPEDIIPLQPVGRMVLNKNIDNFFAE Ac_CatX3
YVKFHWKPTCGVKCLL-DEEAIKVGGSNHSHATKDLYDSIAAGNYPEWKLFIQTIDPDHEDRFD-FDPLDVTKIWPEDILPLQPVGRLVLNRNIDNFFAE Sl_CatX1
YVKFHWKPTCGVKCLL-DEEAIKVGGANHSHATKDLYDSISAGNYPEWKLFIQTIDPDHEDRFD-FDPLDVTKIWPEDILPLQPVGRLVLNKNIDNFFAE St_CatX1
YVKFHWKPTCGVKCLL-EEEAIKVGGANHSHATKDLYDSIAAGNYPEWKLFIQIIDPDHEDRFD-FDPLDVTKTWPEDILPLQPVGRLVLNKNIDNFFAE Np_Cat3
YVKFHWKPTCGVKCLL-DDEAVKVGGTNHSHATQDLYDSIAAGNYPEWKLFIQTIDPDHEDKFD-FDPLDVTKIWPEDILPLQPVGRLVLNKNIDNFFVE Ca_CatX1
YVKFHWKPTCGIKCLS-DEEAIRVGGANHSHATKDLYDSIAAGNYPQWNLFVQVMDPAHEDKFD-FDPLDVTKIWPEDILPLQPVGRLVLNKNIDNFFNE At_CAT1
YVKFHWKPTCGIKCLS-DEEAIRVGGSNHSHATKDLYDSIAAGNYPQWNLFVQVMDPAHEDKFD-FDPLDVTKIWPEDILPLQPVGRLVLNKNIDNFFNE Al_CatX2
YVKFHWKPTCGIKCLL-DEEAIKVGGSNHSHATKDLYDSIAAGNYPQWDLFVQVMDPAHEDKFD-FDPLDVTKIWPEDILPLQPVGRLVLNKNIDNFFNE Cr_CatX1
YVKFHWKPTCGVKSLL-EEEAIRVGGANHSHATQDLYDSIAAGNYPEWKLFIQIMDPEHEDKFD-FDPLDVTKTWPEDILPLQPVGRLVLNKNIDNFFNE Sl_CatX2
YVKFHWKPTCGVKSLL-EEEAIRVGGANHSHATQDLYDSIAAGNYPEWKLFIQIMDPDHEDKFD-FDPLDVTKTWPEDILPLQPVGRLVLNKNIDNFFNE St_CatX2
YVKFHWRPTCGVKSLL-EEEAIRVGGANHSHATQDLYDSIAAGNYPEWKLFIQTMDPAHEDRFD-FDPLDVTKTWPEDIFPLQPVGRLVLNKNIDNFFNE Ca_CatX2
YVKFHWKPTCGVKSLL-EDEAARVGGANHSHATQDLYDSIAAGNYPEWKLFIQTMDPDHEDRFD-FDPLDVTKTWPEDILPLQPVGRLVLNKNIDNFSNE Np_Cat1
YVKFHWKPTCGVKSLL-EEDAIRVGGTNHSHATQDLYDSIAAGNYPEWKLFIQIIDPADEDKFD-FDPLDVTKTWPEDILPLQPVGRMVLNKNIDNFFAE At_CAT2
YVKFHWKPTCGVKSLL-EEDAIRVGGTNHSHATQDLYDSIAAGNYPEWKLFIQIIDPADEDKFD-FDPLDVTKTWPEDILPLQPVGRMVLNKNIDNFFAE Al_CatX1
YVKFHWKPTCGVKSLL-EEDAIRVGGTNHSHATQDLYDSIAAGNYPEWKLFIQIIDPADEDKFD-FDPLDVTKTWPEDILPLQPVGRMVLNKNIDNFFAE Cr_CatX2
YVKFHWKTTSGIKCLL-EEEAIKVGGANHSHATQDLHDSIAAGNYPEWKLFVQTIDPEHEDKFD-FDPLDVTKTWPEDIIPLQPVGRLVLNKNIDNFFAE Gm_CAT4
YVKFHWKTTSGIKCLL-EEEAIKVGGANHSHATQDLHDSIAAGNYPEWKLFVQTIDPEHEDKFD-FDPLDVTKTWPEDIIPLQPVGRLVLNKNIDNFFAE Gm_CAT5
YVKFHWKPTCGVKCLL-EEEAIKVGGANHSHATQDLYDSIAAGNYPEWKLFVQTIDPAHEDKFD-FDPLDVTKTWPEDIIPLQPVGRLVLNKNIDNFFAE Mt_CatX1
YVKFHWKPTCGVKCLL-EEEAIKVGGSNHSHATQDLYDSIAAGNYPEWKLYIQTIDPDHEDKFD-FDPLDVTKTWPEDIIPLQPVGRLVLNKNIDNFFAE Tp_CatX1
YVKFHWKPTCGVKSLL-EEEAIKVGGANHSHATQDLYESIAAGNYPEWKLYIQTIDPDHEDRFD-FDPLDVTKTWPEDIIPLQPVGRLVLNKNIDNFFAE Car_CatX1
YVKFHWKTTCGEKCLL-DDEAIRVGGSNHSHATQDLYDSIAAGNYPEWKLYIQTLDPENEDRLD-FDPLDVTKTWPEDVLPLQPVGRMVLNKNIDNFFAE Gm_CAT3
YVKFHWKTTSGEKSLL-DDEAIRVGGSNHSHATQDLYDSIAAGNYPEWKLYIQTLDPENEDRLD-FDPLDVTKTWPEDVLPLQPVGRMVLNKNIDNFFAE Gm_CAT1/cat2
YVKFHWKPTCGVKNLL-DDEAVVVGGTNHSHATHDLYDSIAAGNYPEWKLFIQTMDPQDEDKFD-FDPLDVTKTWPEDIFPLQPVGRMVLNKNIDNFFAE Gm_CatX1
YVKFHWRPTCGVKCLM-KKDAVMVGGSNHSHATKDLYDSIASGNYPEWKLFIQTINPADEDKYD-FDPLDVTRTWPEDIFPLQPVGRMVLNKNIDNFFVE Sc_CAT2
YVKFHWKPTCGLKYLS-KQEAVVVGGSNHSHATKDLYDSIAAGNYPEWKLFIQTMNPSNEEMFD-FDPLDVTKTWPEEMFPLQPVGRMVLNKNIDNFFAE Sm_CatX2
YVKFHWKPTCGVKSLL-EEQAVIVGGSNHSHATQDLQDSIAAGNYPEWKLFIQTMEIKNENNYD-FDPLDVTKTWPEDLFPLQPVGRLVLNKNIDNFFAE Sm_CatX3
LVKFHWLPSQGVKCLS-PEEAVVVGGTNHSHATQDLTDAIAAGDFPEWKLYIQTMDPATQNNYD-FDPLDVTKVWPEEMFPMQPVGRMVLNKNIDNFFGE Mp_CatX2
YVKFHWKPTCGVKCLI-NDEHIVVGGNNHSHATQDLYDSIAAGNYPEWKLFIQTLDPADEDKFD-FDPLDVTKTWPEDLFPLQPVGRMVLNKNIDNFFAE Sc_CAT1
FVKFHWIPTCGEENLI-GDEAVIVGGTNHSHATKDLYDSIAAGDFPEWKLFIQTLDPADEDKYD-FDPLDVTKTWPEELFPLQPVGRMVLNRNIDNFFAE Ap_CAT5
FVKFHWIPTCGEENLI-GDEAVIVGGTNHSHATKDLYDSIAAGDFPEWKLFIQTLDPADEDKYD-FDPLDVTKTWPEELFPLQPVGRMVLNRNIDNFFAE Ap_CAT8
FVKFHWIPTCGEENLI-GDEAVIVGGTNHSHATKDLYDSIAAGDFPEWKLFIQTLDPADEDKYD-FDPLDVTKTWPEELFPLQPVGRMVLNRNIDNFFAE Ap_CAT1
FVKFHWIPTCGEENLI-GDEAVIVGGTNHSHATKDLYDSIAAGDFPEWKLFIQTLDPADEDKYD-FDPLDVTKTWPEELFPLQPVGRMVLNRNIDNFFAE Ap_CAT3
FVKFHWIPTCGEENLI-GDEAVIVGGTNHSHATKDLYDSIAAGDFPEWKLFIQTLDPADEDKYD-FDPLDVTKTWPEELFPLQPVGRMVLNRNIDNFFAE Ap_CAT4
FVKFHWIPTCGEENLI-GDEAVIVGGTNHSHATKDLYDSIAAGDFPEWKLFIQTLDPADEDKYD-FDPLDVTKTWPEELFPLQPVGRMVLNRNIDNFFAE Ap_CAT6
FVKFHWIPTCGEENLI-GDEAVIVGGTNHSHATKDLYDSIAAGDFPEWKLFIQTLDPADEDKYD-FDPLDVTKTWPEELFPLQPVGRMVLNRNIDNFFAE Ap_CAT7
YVKFHWKPTCGVKSLL-DEEAVMVGGANHSHATQDLYDSIASGNFPEWKIMIQTMNPADEDKFD-FDPLDDTKIWPEDMFPLQPVGRMVLNRNIDNFFAE Pp_CatX3
YVKFHWKPTCGVKNLL-DDEAVLVGGANHSHATQDLYDSIAAGNFPEWKIMIQTMNPADENKFE-FDPLDDTKIWPEDRFPLQPVGRMVLNRNIDNFFAE Pp_CatX6
YVKFHWKPSCGVKFLL-EEEAVIVGGTNHSHMTQDLFDTIAAGNYPEWGLFIQTMEPCMEDAYD-FDPLDDTQTWPEDKFPLQPVGKMVLNKNIDNNFAE Mp_CatX3
FIKWHWKPSCGLKFLL-DEEAIIVGGTNHSHATQDLYENIAAGNYPEWSLYIQTMDPSMEDKVD-FDPLDTTKTWPEDLFPLQPVGKMVLNKNIDNHFAE Mp_CatX4
YVKFHWKPTCGLKFLL-EDEAIVVGGTNHSHATKDLYDTIASGNFPEWKLFIQTMDPATEHQYE-FDPLDDTKTWPEDRFPLQPVGRMVLNKNIDNFFNE Ap_CAT2
YVKFHWKPTCGVSCLM-DDEATLVGGKNHSHATQDLYDSIAAGNFPEWKLFVQVIDPEEEERFD-FDPLDDTKTWPEDEVPLRPVGRLVLNRNVDNFFNE Os_CatA
YVKFHWKPTCGVSCLM-DDEATLVGGKNHSHATQDLYDSIDAGNFPEWKLFVQVMDPDEEDKYD-FDPLDDTKTWPEDLLPLRPVGRLVLDRNVDNFFNE Bd_CatX3
YVKFHWKPTCGVRCILTDEEAALVGGRNHSHATQDLYDSIAAGSFPEWTLYVQVMEPDTEEQYD-FDPLDDTKTWPEDLLPLRPVGRLVLDRNVDNFFNE Zm_Cat3
YVKFHWKPTCGVRSILTDEEAALVGGRNHSHATQDLYDSIAAGSFPEWKLFVQVMDPDTEDQYD-FDPLDDTKTWPEDLLPLQPVGRLVLDKNVDNFFNE Sb_CatX3
YVKFHWKPTCGVKCLM-EDEAAVVCGRNHSHATQDLYDSIAAGIYPEWKLYVQVMDPDHEDQYD-FDPLDDTKTWPEDLLPLQPVGRLVLNRNIDNFFAE Ac_CatX1
YVKFHWKPTCGVKCMS-EEEAIRVGGTNHSHATKDLYDSIAAGNYPEWKLFIQTMDPEDVDKFD-FDPLDVTKTWPEDLLPLIPVGRLVLNRNIDNFFAE Sl_CatX3
YVKFHWKPTCGVKCMS-EEEAIRVGGTNHSHATKDLYDSIAAGNYPEWKLFIQTMDPEDVDKFD-FDPLDVTKTWPEDLLPLIPVGRLVLNRNIDNFFAE St_CatX3
YVKFHWKPTCGVKSMT-EEEAIRVGGTNHSHATKDLYDSIAAGNYPEWKLFIQIMNPEDVDKFD-FDPLDVTKTWPEDILPLMPVGRLVLNRNIDNFFAE Ca_CatX3
YVKFHWKPTCGVKCMT-EEEAIRVGGTNHSHATKDLYDSIAVGNYPEWKLSIHIMDPEDVERFD-FDPLDVTKIWPEDILPLMPVGRLVLNRNIDNFFAE Np_Cat2
FVKFHWKPTCGIKNLT-DEEAKVVGGANHSHATKDLHDAIASGNYPEWKLFIQTMDPADEDKFD-FDPLDVTKIWPEDILPLQPVGRLVLNRTIDNFFNE At_CAT3
FVKFHWKPTCGIKNLT-DEEAKVVGGANHSHATKDLHDAIASGNYPEWKLFIQTMDPADEDKFD-FDPLDVTKIWPEDILPLQPVGRLVLNRTIDNFFNE Cr_CatX3
FVKFHWKPTCGIKNLT-DEEAKVVGGANHSHATKDLHDAISSGNYPEWKLFIQTMDPADEDKFD-FDPLDVTKIWPEDILPLQPVGRLVLNRTIDNFFNE Al_CatX3
LIKWHWRPTCGLKFLL-EDEAAKVVAKCHSHATKDLYDTIASGSFPEWRLYIQTMNPSDEDKYD-FDPMDTTCIWPEELFPLQPVGRMVLNKNLDNFFSE Pp_CatX7
YCKWHWKPTCGLKFLL-DEEAVIVGGTNHSHATKDLYDAIASGNYPEWRIMIQTMDPAQEDRLD-FDPLDTTKIWPEDLFPLQPVGRLVLNRNVDNFFNE Gm_CatX2
LVKYHWEPKQGIKNLT-MKEAAEIQGKNFNHATQDLYEAIERKDYPEWELLVQIMEDGPHPELD-FDPLDDTKLWPEDLFPWRPVGRMVLNKNPENFFNE Pp_CatX8
WVKFHFKTDQGIKNLT-HEEGVKLAGIDPDYAKKDLYNSIEAGDFPSWTFFVQVMSLEQAETFK-WDPFDLTKVWPHKEFPLREVGRFTLDRNPTNYFNE Kf_CatX1
WVKFHFKTNQGIKNWT-HQEAVKVAGESPDSAKEDLYEAIARGEHPSWTFYVQVMSPEQAETFK-WDPFDLTKTWPHKEYPLQEVGRLVLDRNVTNYFNE Kf_CatX4
WVKFHFISDQGIKNLT-HERAVELAGINPDYSTEDLFQNIQNGNGPSWTFKVQVMPLEDAPSYR-WDPFDLTKVWPHADYPLREVGKLKLDRNPTNYFAE Pp_CatX4
WVKFHFISDQGIKNLT-HERAVELAGINPDYSTEDLFQNIQNGNGPSWTFKVQVMPLEDAPSYR-WDPFDLTKVWPHADYPLREVGKLKLDRNPTNYFAE Pp_CatX5
WVKFHFISDQGIKNLT-HERAVELAGINPDYSTEDLFQNIQKGNGPSWTFKVQVMPLEDAASYR-YDPFDLTKVWPHKDYPLREVGKLKLDRNPTNHFAE Pp_CatX2
WVKFHFLSDQGIKNLT-HERAVELAGINPDYSTEDLFQNIQNGNGPSWTFKVQVMPLEDAPQYR-FDPFDLTKVWSHKDYPLREVGKLKFDRNPTNYFAE Pp_CatX1
WVKFHFLSDQGIKNLT-HEQGIKISGINPDYATEDLIQNIEKGNFPSWTFFVQVMPLEDAEKYK-YDPFDLTKVWLHKDYPLREVGKMVLDRNVTNYFAE Mp_CatX1
WVKFHLVTEHGIKNLT-HEEAVSLSGSKPDYATEDLLTSIEEGNFPAWKFYVQVMPLEDAPKYK-WDPFDLTKVWLHKDYPLMEVGRLVLDRNVENYHAE Sm_CatX1
```

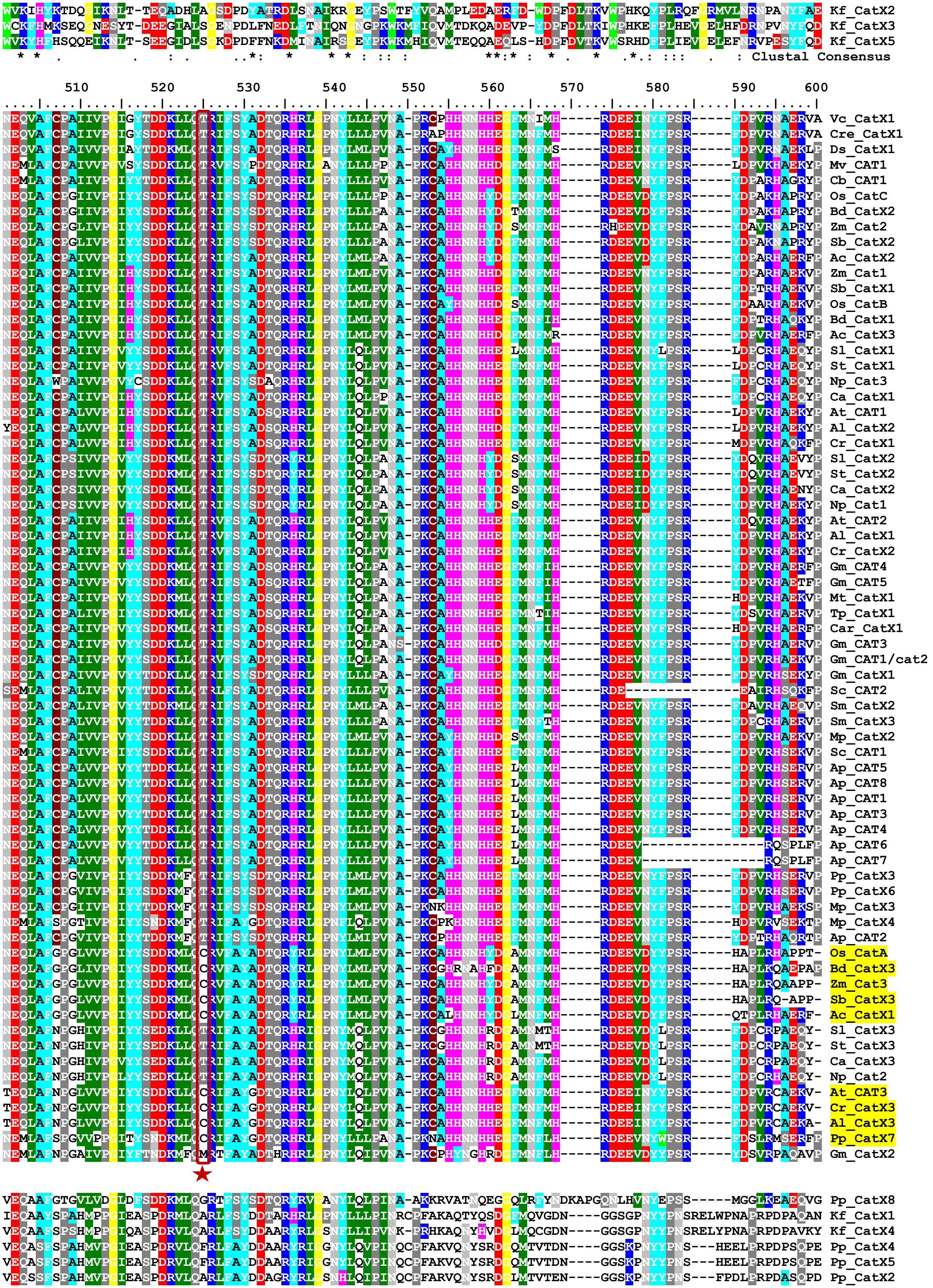
Kf_CatX2
Kf_CatX3
Kf_CatX5
Clustal Consensus
510 520 530 540 550 560 570 580 590 600
Vc_CatX1
Cre_CatX1
Ds_CatX1
Mv_CAT1
Cb_CAT1
Os_CatC
Bd_CatX2
Zm_Cat2
Sb_CatX2
Ac_CatX2
Zm_Cat1
Sb_CatX1
Os_CatB
Bd_CatX1
Ac_CatX3
Sl_CatX1
St_CatX1
Np_Cat3
Ca_CatX1
At_CAT1
Al_CatX2
Cr_CatX1
Sl_CatX2
St_CatX2
Ca_CatX2
Np_Cat1
At_CAT2
Al_CatX1
Cr_CatX2
Gm_CAT4
Gm_CAT5
Mt_CatX1
Tp_CatX1
Car_CatX1
Gm_CAT3
Gm_CAT1/cat2
Gm_CatX1
Sc_CAT2
Sm_CatX2
Sm_CatX3
Mp_CatX2
Sc_CAT1
Ap_CAT5
Ap_CAT8
Ap_CAT1
Ap_CAT3
Ap_CAT4
Ap_CAT6
Ap_CAT7
Pp_CatX3
Pp_CatX6
Mp_CatX3
Mp_CatX4
Ap_CAT2
Os_CatA
Bd_CatX3
Zm_Cat3
Sb_CatX3
Ac_CatX1
Sl_CatX3
St_CatX3
Ca_CatX3
Np_Cat2
At_CAT3
Cr_CatX3
Al_CatX3
Pp_CatX7
Gm_CatX2
Pp_CatX8
Kf_CatX1
Kf_CatX4
Pp_CatX4
Pp_CatX5
Pp_CatX2

```
VEQASFSPGHFVPGIEASPDRVLQARLFAYDDASRYRLGSNYLQIPVNRCPFAKVQTYARDGFMNVSDN----GGSNPNYYPNS---FEDLPRPDPSQPD Pp_CatX1
VEQSAFSPAHMVPGIEASPDRVLQARLFAYDDAARYRLSGNYLQLPINACPFAKVQNYARDGTMVTTDN----GGSKPNYYPNS---FSDLPQPDSAQKD Mp_CatX1
VEQAAFSPGSFVPGIEASPDRILQSRLFAYDDAARYRIGTNFHDVPINK-PIVKVQTYSRDGAMVVSDN----GGSKPNYYPNS---YDNLPQNDPTQPD Sm_CatX1
VEQAAFSPAHMVPGIEPSPDRMLQGRLFSYDDTHRHRLGPNYLQIPINR-PINPQSNHQRDGLMTVNEN----GGAMPNYFPNS---VEGTPAPQPQRPD Kf_CatX2
VEQASFAPANVVPGIGYSPDRMLQARLFSYKDAAVYRLGINHEQLPVNK-PRCPVFNYHSDGFMQTQN------GAGPPYEPNS----FGGPAEDARYVD Kf_CatX3
VEQAAFAPANIVPGLGFSPDRLLQGRLFAYRDAHNHRLGINGFQLPVNQ-PRCPIHTYHRDGAFRMDGN----GGMGPHYEPNS----FGGPQPDPKYAD Kf_CatX5
 *  :: .. .  *:  : *:::* * *:* *:  :*:. *   :* *  .      .    * :                      Clustal Consensus

              610         620         630         640         650         660         670         680         690         700
....|....|....|....|....|....|....|....|....|....|....|....|....|....|....|....|....|....|....|....|
A---VSTAPLSGRREKVVIAKENNFAQAGARWRSFDPARQERLVVRVADMLADP--------RCTQEIRRIWVGYWSQADPQLGSRIAAKLQGRGCL--- Vc_CatX1
A---VSSAPLSGRREKMVIAKENNEMQAGARWRAFDAARQERFVVRVADMLADP--------RCTQEIRRIWVGYWSQADPQLGARIAAKLQGRGCL--- Cre_CatX1
V---HSSAHISGKREKVVIAKENNFQQAGERFRSFDPARQERFVGRAAGFLMEP--------KCTNEIRRIWVGYWSQCDASLGQRIAAKLRQAGAL--- Ds_CatX1
----FSPQVISGVRTKAVIEKENNFKQPGERYRAMDPARKARFVGRLADSLSCP--------RIPLETRRTVVGYWAQADAGLGQELARRLKMTPTM--- Mv_CAT1
----IPHEIVHGVREKAVISKENNFQQPGERYRSWAPERQERFVNRMASALSDP--------RVTHEIRGVWISYLSQADQSLGMKVAAKLGMKASL--- Cb_CAT1
----IPSATLTGRREKVVIAKENNFKQPGERYRSWDPARQDRFIKRWIDALSDP--------RLTHEIRSIWLSYWSQADRSLGQKLASRLSAKPSM--- Os_CatC
----IPSSTCNGRREKMVIEKENNFKQPGERYRSMDPARQERFINRWIDALSDP--------RLTHEIKSIWLSYWSQADRSLGQKLASRLSAKPSM--- Bd_CatX2
----IPTAHIAGRREKTVISKENNFKQPGERYRAMDPARQERFITRWVDALSDP--------RLTHEIRTIWLSNWSQADRSLGQKLASRLSAKPSM--- Zm_Cat2
----IPAVHITGRRDKTVIAKENNFKQPGERYRAMDPARQERFIKRWVDALSDP--------RLTHEIRSIWLSNWSQADRSLGQKLASRLSAKPSM--- Sb_CatX2
----IPPRILTGRREKCVIEKENNFKQPGERYRSFDPARQERFIKRWVDALSDP--------RVTHEIRSIWISYWSQCDKSLGQKLATRLNLKPSM--- Ac_CatX2
----IPPRVLTGCREKCIIQKENNFKQAGERYRSFDPARQDRFIQRWVDALTDP--------RVTHEHRTIWISYWSQCDAALGQKLASRLNLKPSM--- Zm_Cat1
----IPSRVLTGCREKCIIEKENNFKQAGERFRSFDPARQDRFIQRWVDALSDP--------RVTHEHHTIWINYWSQCDASLGQKLASRLNLKPNM--- Sb_CatX1
----IPPRVLTGCREKCVIDKENNFQQAGERYRSFDPARQDRFLQRWVDALSDP--------RITHELRGIWISYWSQCDASLGQKLASRLNLKPNM--- Os_CatB
----IPTRVLTGCREKCIIEKENNFKQAGERYRSFDPARQDRFIQRWVDALSDA--------RVTHEIQGIWISYWSQCDTSLGQKLGSRLKAKPNM--- Bd_CatX1
----IPPCVLSGRRERCVIEKENNFKQPGERYRSFDPDRQERFIQRWVDALTDP--------RVTHELRSIWISYWSQCDQSLGQKLATRLSVKPNM--- Ac_CatX3
----IPPRVLTGKREKVIIEKENNFKQPGEHYRSWAPDRQERFLCRWVDALSDP--------RATHEIRSIWISYWSQADKSLGQKLASRLNIRPTM--- Sl_CatX1
----IPPRVLTGKREKVIIEKENNFKQPGEHYRSWAPGRQERFVCRWVDALSDL--------RATHEIRSIWISYWSQADKSLGQKLASRLNIRPTM--- St_CatX1
----IPPCVLTGKRDKCIIEKENNFKQPGERYRSWAPDRQERFICRWVDALSDP--------RVTHEIRSIWFSYWSQADKTLGQKIASRLNVRPTM--- Np_Cat3
----IPPRVLNGKRDRCIIQKENNFKQPGERYRSWAPDRQERFICRWVDALSEP--------RVTHEIRTIWLSYWSQADKSLGQKLASRLNVKPTM--- Ca_CatX1
----TTPIVCSGNREKCFIGKENNFKQPGERYRSWDSDRQERFVKRFVEALSEP--------RVTHEIRSIWISYWSQADKSLGQKLATRLNVRPNF--- At_CAT1
----TTPIVCTGNREKCFIGKENNFQQPGERYRSWDSDRQERFVKRFVEALSEP--------RVTHEIRSIWISYWSQADKSLGQKLATRLNVRPNF--- Al_CatX2
----ATPIICSGNREKCIIGKENNFKQPGERYRSWDSDRQERFLKRFVEALSEP--------RVTHEIRSIWISYWSQADKSLGQKLATRLNVRPNI--- Cr_CatX1
----IPSTVCSGKREKCIIQKENNFKQPGERYRSFTPDRQERFIRRWVEALSDP--------RITYEIRSIWITYWSQADKSLGQKLASRLNVRPSI--- Sl_CatX2
----IPSTVCSGKREKCIIQKENNFKQPGERYRTFTPDRQERFIRRWVEALSDP--------RITYEIRSIWITYWSQADKSLGQKLASRLNVRPSI--- St_CatX2
----IPTTVCSGKREKCIIQKENNFQQPGERYRSFSADRQERFIRRWVEALSDP--------RITYEIRSIWISYWSQADKSLGQKLASRLNVRPSI--- Ca_CatX2
----IPSTMCTGKREKCVIQKENNFKQPGERYRSFTPDRQERFIRRWVETLSDP--------RITYEIRSIWISYWSQADKSLGQKLASRLNVRPSI--- Np_Cat1
----TPPAVCSGKRERCIIEKENNFKEPGERYRTFTPERQERFIQRWIDALSDP--------RITHEIRSIWISYWSQADKSLGQKLASRLNVRPSI--- At_CAT2
----TPPAVCSGKRERCIIEKENNFKEPGERYRSFTPERQERFIQRWIEALSDP--------RITHEIRSIWISYWSQADKSLGQKLASRLNVRPSI--- Al_CatX1
----TPPAVCSGKRERCVIEKENNFKEPGERYRSFPPERQERFIRRWIEALSDP--------RITHEIRSIWISYWSQADKSLGQKLASRLNVRPSI--- Cr_CatX2
----IPPAICSGRREKCGIEKENNFKQPGERYRSWAPDRQDRFARRWVDALSDP--------RVTHEIRSVWISYWSQADRSLGQKIASHLSTRPNI--- Gm_CAT4
----IPPAICSGRREKCGIEKENNFKQPGERYRSWAPDRQDRFNRRWVDALSDP--------RVTHEIRSVWISYWSQADRSLGQKIASRLSMRPNI--- Gm_CAT5
----IPTANFSACREKCNIPKQNNFKQPGERYRSWAPDRQDRFLRRWVDALSDP--------RVTHEIRSIWISYWSQADRSLGQKIASHLNMRPSI--- Mt_CatX1
----IPTPTLSARREKTNIPKQNNFKQPGERYRSWPADRQDRFLRRWVDALSDP--------RVTHEIRSIWISYWSQADRSLGQKIASHLNMRPSI--- Tp_CatX1
----TPPAALSGKRERCIRPKENNFKQPGERYRTWAPDRQDRFARRWIEALSDP--------RVTHEVRSIWISYWSQADRSLGQKIASHLNMRPSI--- Car_CatX1
----VPPRTLGGKREKCMIEKENNFKQPGERYRSWPSDRQERFVRRWVDALSDP--------RVTHEIRSIWISYWSQADRSLGQKIASHLNLKPSI--- Gm_CAT3
----VPPRILGGKREKCMIEKENNFKQPGERYRSWPSDRQERFVRRWVDALSDP--------RVTHEIRSIWISYWSQADRSLGQKIASHLNLKPSI--- Gm_CAT1/cat2
----VPSHDVNGQREKCIIEKENNFKQAGERYRSFASDRQDRFVTRWVNALSDP--------RVTPEILSIWLHYWSQCDKSLGQKIANRLNTKASM--- Gm_CatX1
----TPSNTLMGRREKVVIEKENNFVEPGRRYRSWDPERQDRFVNVWVEALSDP--------RVTHEVRDVWLSYLSQADQSLGQKVAKCLNMKSNIVVV Sc_CAT2
----IPSRPVSGRREKVMLEKENNFAQPGVRYRSWPADRQERFIENWVDALSDS--------RVTHEIRSVWISYWSQCDKSLGQRIAAQLQSKSNM--- Sm_CatX2
----IPSHPLGGRREKVMIEKENNFAQPGARFRSWPADRQERFIARVVDALSDP--------RVTHEIRSIWLSYWSQADRMLGQKIATKLNVKANM--- Sm_CatX3
----IPSHVLTGRREKVMIEKENNFAQPGARYRSWAPDRQERFVKQWVDNLSDA--------RVTQEVRNIWLGYWAQCDKSLGQKLAS--RVKVTSSM- Mp_CatX2
----IPSIVLRGRREKVMIEKENNFSQPGARYRSWAPDRQERFVKMWVDMLSNS--------RVTHEIRSIWLSYWSQADRSLGQKISAGLQRKLKSNM- Sc_CAT1
----IPSAPLSGKREKVSDEE----------LREIAERAGEVKDVLEVDFPFEA--------CCTRRLPCEGVAAF-------------------------- Ap_CAT5
----IPSAPLSGKREKVSDEE----------LREIAERAGEVKDVLEVDFPFEA--------CCTRRLPCEGVAAF-------------------------- Ap_CAT8
----IPSAPLSGKREKVMIEKENNFQQPGVRYRSWAPDRQERFVNMWVDALSDP--------RVTHEIRSIWISYWSQVR-----KHSRNLLS-------- Ap_CAT1
----IPSAPLSGKREKVMIEKENNFQQPGVRYRSWAPDRQERFVNMWVDALSDP--------RVTHEIRSIWISYWSQCDGSLGQKIAAGLNSKARL--- Ap_CAT3
----IPSAPLSGKREKVMIEKENNFQQPGVRYRSWAPDRQERFVNMWVDALSDP--------RVTHEIRSIWISYWSQVR-----KHSRNLLS-------- Ap_CAT4
----VEIM-FSGKRP-----------------------------------LHHPVFF--------LVAGKLLPI--------------------------- Ap_CAT6
----VEIM-FSGKRP-----------------------------------LHHPVFF--------LVAGKLLPI--------------------------- Ap_CAT7
----VSQSRLLGIREKAVIHKENNFQQPGVRFRSFDPARQDRFIKRIIELLSDP--------RVTHEVRSVWVAYWSQCDKTLGQRIASHFS---MKAAM Pp_CatX3
----ISQSRLAGVREKAVIPKENNFQQAGERFRSFDPARQDRFIKRIVELLSDP--------RVTQEVRSVWFSYWMQCDRTLGQRIASQFS---MKASM Pp_CatX6
----LPQNSFSGKREKAIIKKENNFKQAGERYRTFDPARQERFLMRFVGVLSDP--------KVTDEVRKAWVFYWTQADASLGNKLAAKLNMPAIQSGM Mp_CatX3
----ISQDIVSGKREKVVIYKENNFKQPGVLYRTFDTARQERFIKRFVDSLKDP--------RVTDEVRKVWVYYCAQADQTLGKKMAQRLNMPMIQSGM Mp_CatX4
----VPRNQVSGVRDKINIEKENNFQQPGERYRSWPSDRQDRFVERWVKALSDP--------KVTHEIRSIWISYWSQCDKGLGQKLTSRLN---VRSAI Ap_CAT2
---PITPRPVVGRRQKATIHKQNDFKQPGERYRSWAPDRQERFIRRFAGELAHP--------KVSPELRAIWVNYLSQCDESLGVKIANRLNVKPSM--- Os_CatA
ACFPVPVRTVVGKREKTRIKKENDFQQSGERYRSWAPDRQDRFVKRFADALGHP--------KVSHELRAIWVNFLSQCDKSCGMKVANRLNVKPSM--- Bd_CatX3
--TPLPPRPVAGRREKATIRKPNDFKQPGERYRSWDADRQDRFVRRFADSLGHP--------KVSQELRSIWIDLLAKCDASLGMKIATRLNMKANM--- Zm_Cat3
--VPIPARPVVGKRQKETIRKPNDFKQPGERYRSWDADRQERFVRRFADSLGHP--------KVSQELRSIWIDLLSKCDASLGMKIATRLNMKPNM--- Sb_CatX3
---PIPTRPVTGRREKITIHKQNDFKQPGERYRSWTPDRQDRFVRRWAEALGHP--------KVSHELRSIWISYLSQCDTSLGMKVANRLNVKPSM--- Ac_CatX1
---PIPSCVLNGRRTNCVIPKENNFKQAGERYRSWEPDRQDRYINKWVESLSDP--------RVTHEIRSIWISYLSQADKSCGQKVASRLTVKPTM--- Sl_CatX3
---PIPACVLNGRRTNCVIPKENNFKQAGERYRSWESDRQDRYITKWVESLSDP--------RVTHEIRSIWISYLSQADKSCGQKVASRLTVKPTM--- St_CatX3
---PIPSCVLTGRREKCVIPKENNFKQAGERYRSWAPDRQDRYINKWVESLSDP--------RVTHEIRSIWISYLSQADKSCGQKVASRLTVKPTM--- Ca_CatX3
---PIPSRVLNGRREMCVIEKENNFKQAGERYRSWEPDRQDRYVSKWVEHLSDP--------RVTYEIRSIWISYLSQADKSCGQKVASRLTLKPTM--- Np_Cat2
---PTPTNSYTGIRTKCVIKKENNFKQAGDRYRSWAPDRQDRFVKRWVEILSEP--------RLTHEIRGIWISYWSQADRSLGQKLASRLNVRPSI--- At_CAT3
---PIPTNSYTGIRTKCVIKKENNFKQAGDRYRSWAPDRQDRFVKRWVEILSEP--------RLTHEIRGIWISYWSQADRSLGQKLASRLNVRPSI--- Cr_CatX3
---PIPTNSYTGIRTKCVIKKENNFKQAGDRYRSWAPDRQDRFVKRWVEILSEP--------RLTHEIRGIWISYWSQV-RTKNHSVKSLLPFSPNIQQT Al_CatX3
----ISKAHVSGIREKTVIPKENNFIQPGNRYRTWDHDRQERFIGRMVTALSDP--------RLTHEIRAIWISYMSQCDKTLGQKLASRLSTRATM--- Pp_CatX7
----LPRDTVTGRRERTVIPKENNFKQAGEMFRTWDSDRQERFIKRFADAVSDP--------KVTAEVRSIWLGFWTQCDRSLGQRLAARLNSRSFY--- Gm_CatX2
---TEYTPHVEGNLVRESIDRQSNTKQAGDTYRHFEQWEKDELINNLVNDLA---------ICPQVIQDKMVALAEEADEDYGRRLRAGLMEKSKMKDG Pp_CatX8
PEPFPVEGGLAGRYPNKKWVDQNDWDQPQMLWKTMSKEDQEQTLSNIAGHLQG---------AQEFIQKRAIALFGKIDPEMEEGLQAILPEKKKSDEP Kf_CatX1
PEPFQVPAGLAGRYINKKWADQDDWDQPRALWEKFSDEDKEQTLSNIVGMLAP---------CQQFLQDRVVGLFGKIHEDMAAGLTKRLNAAKEGKAK Kf_CatX4
VHPYEHAGGTVSKAPPRKWSEQDDYEQPRMLWQKMSKEDQGQTAKNIAGHIQG---------AKESIIERQMGVFRRCDPSLADKVEEMLGMKKKSEDP Pp_CatX4
VHPYEHAGGTVSKAPPRKWSEQDDYEQPRMLWQKMSKEDQGQTAKNIAGHIQG---------AKESIIERQMGVFRRCDPSLADKVEEMLGMKKKSEDP Pp_CatX5
```

```
VHPYKHEGGIVSRTPPRKWSMQDDYEQPRALWQTMSKEDQGQTAKNIAGHIQG----------AQEFIIQRQMGIFKRCDPSLAQKVEEMLEMKRKSQDP Pp_CatX2
VHPYKHDGGVVSRTPPRKWTEQDDYEQPRALWQAMSAQDRGQTAKNIAGHIMG----------AKEMIIQRQMGVFRKCDESLASKVEEMLAVKRIGQDP Pp_CatX1
VHPYNHAAGVVDRKPARRWVEDDDYEQPRMLWQNMSSEDQEQTAKNIAGHIMG----------AKDFIQKRQIEVFRRCDEKLADKVEEALAEKKKSSEP Mp_CatX1
VHP--HEGGTVGRFPPRKWEEQDDYEQPRMLWKLMSEEDKEQTVSNIANHIRG----------AAEFIQERQMGLFRKCDGDLASRIEKKLAEKKKSPEP Sm_CatX1
VHMYQSKGGAIGRKVFKKWQEQDDYSQPGALFRLMPKNEQDQLIANLAGHMSA----------AKEHIRRREIEVFRRCDAELGRRLDQAIADKVRGASH Kf_CatX2
PAVPLQMSGEGKRYNHR--ADSDDYSQATALWHIMKPDEQERVITGITNTMTKGKGAGCKSDPVPKKIQQLNLYHYYRVDKSLGERLAESLGLDLKETIA Kf_CatX3
PAIPINVTGPGMRYENH--YGKDDFMHAKAMWEMFEPDEQERVIANMARSMKEGTEAGVNSEPVPELIQQINLYNFARVDEDLGNKLAAALGLDLKSSMD Kf_CatX5
                                                                                            Clustal Consensus

           710        720        730
....|....|....|....|....|....|....
-----------------------------------                                                                      Vc_CatX1
-----------------------------------                                                                      Cre_CatX1
-----------------------------------                                                                      Ds_CatX1
-----------------------------------                                                                      Mv_CAT1
-----------------------------------                                                                      Cb_CAT1
-----------------------------------                                                                      Os_CatC
-----------------------------------                                                                      Bd_CatX2
-----------------------------------                                                                      Zm_Cat2
-----------------------------------                                                                      Sb_CatX2
-----------------------------------                                                                      Ac_CatX2
-----------------------------------                                                                      Zm_Cat1
-----------------------------------                                                                      Sb_CatX1
-----------------------------------                                                                      Os_CatB
-----------------------------------                                                                      Bd_CatX1
-----------------------------------                                                                      Ac_CatX3
-----------------------------------                                                                      Sl_CatX1
-----------------------------------                                                                      St_CatX1
-----------------------------------                                                                      Np_Cat3
-----------------------------------                                                                      Ca_CatX1
-----------------------------------                                                                      At_CAT1
-----------------------------------                                                                      Al_CatX2
-----------------------------------                                                                      Cr_CatX1
-----------------------------------                                                                      Sl_CatX2
-----------------------------------                                                                      St_CatX2
-----------------------------------                                                                      Ca_CatX2
-----------------------------------                                                                      Np_Cat1
-----------------------------------                                                                      At_CAT2
-----------------------------------                                                                      Al_CatX1
-----------------------------------                                                                      Cr_CatX2
-----------------------------------                                                                      Gm_CAT4
-----------------------------------                                                                      Gm_CAT5
-----------------------------------                                                                      Mt_CatX1
-----------------------------------                                                                      Tp_CatX1
-----------------------------------                                                                      Car_CatX1
-----------------------------------                                                                      Gm_CAT3
-----------------------------------                                                                      Gm_CAT1/cat2
-----------------------------------                                                                      Gm_CatX1
LCKLDTGT---------------------------                                                                      Sc_CAT2
-----------------------------------                                                                      Sm_CatX2
-----------------------------------                                                                      Sm_CatX3
-----------------------------------                                                                      Mp_CatX2
-----------------------------------                                                                      Sc_CAT1
-----------------------------------                                                                      Ap_CAT5
-----------------------------------                                                                      Ap_CAT8
-----------------------------------                                                                      Ap_CAT1
-----------------------------------                                                                      Ap_CAT3
-----------------------------------                                                                      Ap_CAT4
-----------------------------------                                                                      Ap_CAT6
-----------------------------------                                                                      Ap_CAT7
-----------------------------------                                                                      Pp_CatX3
-----------------------------------                                                                      Pp_CatX6
-----------------------------------                                                                      Mp_CatX3
-----------------------------------                                                                      Mp_CatX4
-----------------------------------                                                                      Ap_CAT2
-----------------------------------                                                                      Os_CatA
-----------------------------------                                                                      Bd_CatX3
-----------------------------------                                                                      Zm_Cat3
-----------------------------------                                                                      Sb_CatX3
-----------------------------------                                                                      Ac_CatX1
-----------------------------------                                                                      Sl_CatX3
-----------------------------------                                                                      St_CatX3
-----------------------------------                                                                      Ca_CatX3
-----------------------------------                                                                      Np_Cat2
-----------------------------------                                                                      At_CAT3
-----------------------------------                                                                      Cr_CatX3
KHYNLSYLSG-------------------------                                                                      Al_CatX3
-----------------------------------                                                                      Pp_CatX7
-----------------------------------                                                                      Gm_CatX2
FKMEIGEEMHPLGARDAHQAVKDAIEKGHDQDPY                                                                       Pp_CatX8
YTMTA------------------------------                                                                      Kf_CatX1
TSQTA------------------------------                                                                      Kf_CatX4
YTMVA------------------------------                                                                      Pp_CatX4
```

```
YTMVA-----------------------------          Pp_CatX5
YTMVA-----------------------------          Pp_CatX2
YTMVA-----------------------------          Pp_CatX1
YTMVA-----------------------------          Mp_CatX1
YTMIA-----------------------------          Sm_CatX1
VSVNAGNFAVANL---------------------          Kf_CatX2
RAEKDLPKEVPALVSL------------------          Kf_CatX3
VGRKTLEQLYSRQTAGTHKDLSPES---------          Kf_CatX5
                                       Clustal Consensus
```
